# Health-related quality of life in patients with conditions affecting the hand: meta-analysis

**DOI:** 10.1093/bjs/znae067

**Published:** 2024-04-09

**Authors:** Luke Geoghegan, Maria Carolina, James French, Conrad J Harrison, Jeremy N Rodrigues

**Affiliations:** Department of Surgery and Cancer, Imperial College London, London, UK; Department of Orthopaedic Surgery, University of Verona, Verona, Italy; University of Birmingham Medical School, University of Birmingham, Birmingham, UK; Nuffield Department of Orthopaedics, Rheumatology and Musculoskeletal Sciences, University of Oxford, Oxford, UK; Warwick Clinical Trials Unit, University of Warwick, Warwick, UK; Department of Plastic, Reconstructive and Hand Surgery, Stoke Mandeville Hospital, Aylesbury, UK

## Abstract

**Background:**

Health state utility values provide the quality component of quality-adjusted life years and are essential for health economic analyses, such as the National Institute for Health and Care Excellence Technology Appraisal. The aims of this systematic review were to: catalogue utility values for health states experienced by patients with hand conditions; provide pooled utility estimates for common hand conditions; and determine how utilities have been estimated.

**Methods:**

A PRISMA-compliant systematic review and meta-analysis was conducted (registered in PROSPERO, the international prospective register of systematic reviews (CRD42021226098)). Five databases were searched from inception until April 2023 (Embase, MEDLINE, PsycINFO, the Cumulative Index to Nursing and Allied Health Literature (CINAHL), and the Cochrane Central Register of Controlled Trials (CENTRAL)). All studies that reported primary utility values for hand health states in adult patients were eligible for inclusion. Pooled utility estimates were determined across conditions and intervention status using random-effects meta-analysis.

**Results:**

A total of 10 254 articles were identified; 57 studies met the full inclusion criteria and reported 363 distinct health state utility values. Health state utility values were estimated using a range of methods; the most common measure was the EQ-5D. Pooled utility estimates for carpal tunnel syndrome and hand osteoarthritis before surgical intervention were 0.69 (95% c.i. 0.66 to 0.73) and 0.63 (95% c.i. 0.60 to 0.67) respectively.

**Conclusion:**

Pooled utility estimates for patients with untreated carpal tunnel syndrome and hand osteoarthritis are 11% and 18% lower than age-matched population norms respectively. Hand conditions have a significant detrimental impact on health-related quality of life and this study provides catalogued utility values for use in future economic analyses to support the delivery of value-based hand surgery.

## Teaser

This meta-analysis aimed to determine health state utility values for electively managed conditions affecting the hand. Pooled health state utility value estimates for carpal tunnel syndrome and hand osteoarthritis before surgical intervention were 0.69 (95% c.i. 0.66 to 0.73) and 0.63 (95% c.i. 0.60 to 0.67) respectively. These health state utility value estimates suggest patients with untreated carpal tunnel syndrome and hand osteoarthritis have substantial impairments in quality of life and are comparable to health states experienced by patients with coronary artery disease and chronic obstructive pulmonary disease.

## Introduction

Value-based healthcare has emerged to bridge the gap between population demand and available resource. Value in healthcare is defined as outcomes achieved per unit cost^1^. Looking beyond fiscal impact, it may be morally and philosophically justified to incentivize improved outcomes with higher reimbursement and de-incentivize interventions that are not cost-effective from a utilitarian perspective. A range of measures are used to determine value, including cost-effectiveness, cost-utility, and cost-benefit.

Cost-utility analysis is the most common type of economic evaluation and is used by the National Institute for Health and Care Excellence (NICE) in health technology appraisal^2^. Cost-utility analyses assess the value of interventions using quality-adjusted life years (QALYs). QALYs provide a generic measure of intervention effect that encompass longevity and quality of life in a single value. Utility represents the desirability of a health state and forms the quality-adjusted component of QALYs. Utilities are typically expressed on a scale from 0 (equivalent to death) to 1 (equivalent to full health), where negative values denote a health state deemed worse than death^3^.

Utilities for a given health state can be determined through direct preference elicitation techniques, such as standard gamble (SG) and time trade-off (TTO). Utilities can also be determined indirectly using preference-based patient-reported outcome measures (PROMs). A PROM is said to be preference based when the health states described by the PROM have been valued by a population^4^. The EQ-5D is an example of a generic preference-based PROM^5^. The value set associated with the EQ-5D enables health state utility values (HSUVs) to be determined directly from PROM response scores. An individual with moderate problems walking, severe difficulty performing self-care, severe problems performing usual activities (work and hobbies), and severe pain has a utility score of 0.25 when measured with the EQ-5D-5L and associated UK value set.

Utility values are used by agencies, such as NICE, to determine the cost-effectiveness of interventions, populate decision analytic models, and ultimately inform resource allocation. Utilities can vary between populations and by derivation method, meaning a range of utility values can exist for a single health state^6^. For example, patients with high functional demand, such as concert pianists, may assign greater significance to changes in hand function associated with carpal tunnel syndrome (CTS) compared with elderly patients with low functional demand. In this situation, the underlying health state (moderate CTS) is identical, but HSUVs can be markedly different depending on the valuing population.

This is of particular relevance for conditions affecting the hand, where interventions for CTS, Dupuytren’s disease, and trigger finger have been deemed ‘procedures of limited clinical value’^7^. Conditions affecting the hand are common; the estimated prevalence of CTS is 5%^8^, whilst the estimated prevalence of hand osteoarthritis is 41%^9^. Hand conditions can be functionally debilitating, limiting an individual’s ability to work and perform basic activities of daily living^10,11^. Currently, there is no clear overview of utility values for conditions affecting the hand and wrist. Accurate assessment of the value of interventions for hand conditions is necessary to compare the cost-effectiveness of interventions and guide treatment reimbursement to support the delivery of value-based care.

The aims of this systematic review were to: identify and catalogue all utility values for health states experienced by patients with conditions affecting the hand and wrist in the current literature; provide pooled estimates of utility values for each identifiable health state; and determine the impact of derivation methods and populations on utility estimates.

## Methods

This systematic review and meta-analysis followed the PRISMA reporting guideline^12^ and was registered in PROSPERO, the international prospective register of systematic reviews (CRD42021226098). The PRISMA checklist is available as *Supplementary material*. The design and conduct of this systematic review followed contemporary practical guidelines^13^.

### Eligibility criteria

Studies were included if they reported an original utility value for a specified health state experienced by adult patients (aged greater than or equal to 18 years) with conditions affecting the hand and/or wrist (irrespective of primary pathology, intervention status, or type of intervention). Health states were defined as perceivable descriptions comprising pathology, condition severity (where appropriate), intervention status, and the presence of complications (for example, moderate CTS complicated by complex regional pain syndrome after open decompression). Systemic conditions with secondary effects on the hand (such as scleroderma) were excluded. Studies that reported unfeasible utility values (that is values greater than 1) were also excluded.

### Search strategy and study selection

Five databases were searched from inception until April 2023 (Embase, MEDLINE, PsycINFO, Cumulative Index to Nursing and Allied Health Literature (CINAHL), and the Cochrane Central Register of Controlled Trials (CENTRAL)). No limitations were placed on publication date, language, or country of origin. References of included studies were hand searched to identify further potentially eligible articles.

A bespoke search strategy was devised in conjunction with a search strategist. The search comprised index and free-text terms relevant to the following key concepts: hand/wrist/fingers, quality of life, QALYs, and HSUVs. Detailed search terms are provided in *Table S3*.

All identified articles underwent title and abstract screening, followed by full-text review using the Covidence review management platform^14^. Two reviewers performed abstract screening and full-text review in duplicate; conflicts were resolved through consultation with a third reviewer.

### Data extraction

Data were extracted independently by two reviewers using a pre-designed data collection pro forma. Data extracted from eligible studies included author, publication year, country, primary pathology (if applicable), health state described, HSUV mean and/or median, HSUV standard deviation and/or range/interquartile range (i.q.r.), study type, valuation method, associated value set (applicable only for indirect valuation techniques, such as the EQ-5D), respondents (that is the specific population valuing described health states), and number and age of respondents. Utility values for distinct cohorts within a single study population were extracted as separate entities provided the health states of the study populations described were distinct. For example, Atroshi *et al*.^15^ conducted a prospective cohort study that investigated outcomes after endoscopic carpal tunnel release. Utility values were reported at baseline and 3 months after the intervention and therefore utility values were extracted for each health state (that is baseline CTS and 3 months after endoscopic decompression). Risk-of-bias assessments were not performed for included studies, as the present study did not seek to determine intervention effect. At the time of publication, no guidelines for utility derivation studies have been published.

### Statistical analysis

Descriptive statistics are used to present study characteristics and demographics. Utility values for defined health states are summarized narratively and are stratified by primary pathology, valuation technique, and respondent characteristics. Where multiple utility estimates were identified for a defined health state, pooled utility estimates were obtained using random-effects meta-analysis. Pooled utility estimates were only obtained from sufficiently homogeneous health states with adequate availability of primary data, as determined through consensus discussion between three reviewers. Where not reported, mean utility values and associated standard deviations were calculated using reported median utility values and associated range/i.q.r.^16^.

Random-effects meta-analysis was used to obtain pooled mean utility estimates with associated 95% confidence intervals. Random-effects meta-analysis relaxes the assumption that variation between utility estimates between cohorts and studies is solely due to random error. The restricted maximum likelihood (REML) method was used to estimate the heterogeneity of variance due to the small sample sizes in eligible studies^17^. Meta-regression has been used to explore potential sources of heterogeneity^18^; this was not conducted due to the small number of studies included in the pooled analyses and the relatively large number of covariates^19^. All analyses were conducted in the R statistical computing environment (version 4.3.1)^20^ using the Metafor package (version 4.2-0)^21^.

## Results

### Search results

**Fig. 1:**
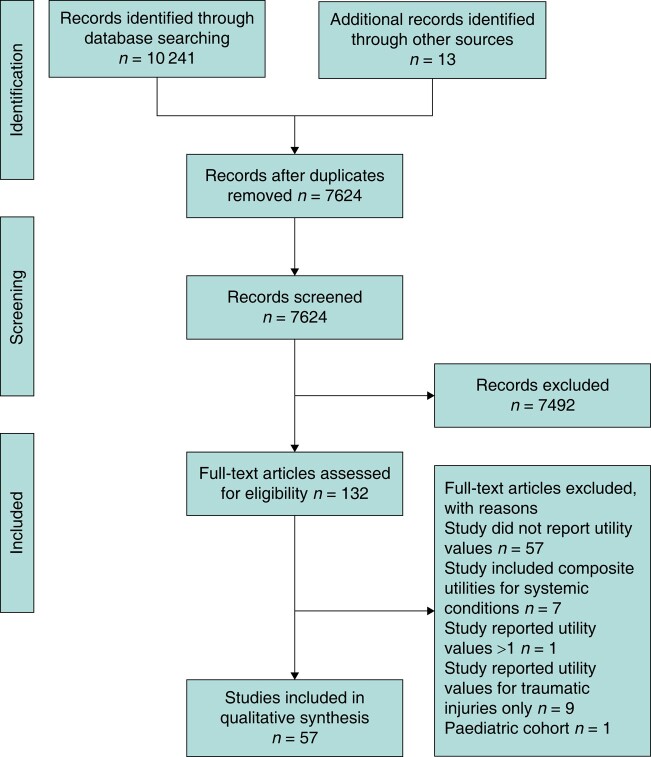
PRISMA flow chart

An initial search identified 8748 studies; a further 13 studies were identified through hand searching reference lists of identified articles. A second search identified a further 1493 studies. A total of 57 studies were included after de-duplication and stepwise screening (*Fig. 1*).

### Study characteristics

The 57 included studies reported 34 590 utility estimates for 363 distinct health states. Included studies were published between 1998 and 2023. Studies were conducted in Europe (56%, 32 of 57), America (28%, 16 of 57), Asia (14%, 8 of 57), and both Asia and America (2%, 1 of 57). Identified studies included 9 RCTs, 29 cohort studies (18 prospective and 11 retrospective), 11 economic evaluation studies, 5 utility derivation/decision analysis studies, and 3 cross-sectional studies. The full reference list of included studies is available on request.

Included studies used direct (47%, 169 of 363) and indirect (53%, 194 of 363) valuation techniques to estimate HSUVs. Direct valuation techniques included TTO (18%, 65 of 363), visual analogue scale (VAS; 18%, 65 of 363), SG (10%, 36 of 363), and chained standard gamble (CSG; 1%, 3 of 363) (*Table S1*). Indirect valuation techniques included the EQ-5D (45%, 164 of 363), the Short-Form 6D (SF-6D; 7%, 24 of 363), the Health Utilities Index-3 (HUI-3; 1%, 4 of 363), and the Health Utilities Index-2 (HUI-2; 1%, 2 of 363) (*Table S2*).

### Health states

HSUVs were estimated by 13 467 individuals. The majority of the 363 health states described were valued by patients (80%, 289 of 363). Health states were also valued by members of the public (10%, 36 of 363) and healthcare professionals (including medical students, nurses, and doctors; 10%, 38 of 363). Health states were thematically grouped by underlying condition/pathology (*Table 1*). The most common condition across identified health states was upper extremity amputation (31%, 113 of 363), followed by CTS (20%, 74 of 363) and hand osteoarthritis (20%, 72 of 363). A number of distinct health states were observed for each core condition due to differences in condition severity, intervention status (before/after), intervention type, time since intervention, and the presence of complications. Catalogued utility values for all health states identified are presented in *Tables S1*, *S2*. *Table S1* presents HSUVs derived using direct valuation techniques; the number of respondents indicating the number of individuals valuing the health state described for each primary study. *Table S2* presents HSUVs derived using indirect valuation techniques (patients valuing their own health states, typically as part of a clinical study); the number of respondents indicating the number of patients in the health state described for each primary study.

**Table 1:** Characteristics of identified health states

	Value
**Condition**	
Upper extremity amputation	113 (31)
Carpal tunnel syndrome	74 (20)
Hand osteoarthritis	72 (20)
Rheumatoid arthritis	50 (14)
Dupuytren’s disease	26 (7)
Various	16 (4)
Hand-arm vibration syndrome	4 (1)
Complex regional pain syndrome	3 (1)
Trigger finger	3 (1)
Scapholunate instability	2 (1)
**Utility instrument**	
Direct measures	
TTO	65 (18)
VAS	65 (18)
SG	36 (10)
C-SG	3 (1)
Indirect measures	
EQ-5D	164 (45)
SF-6D	24 (7)
HUI-2	2 (1)
HUI-3	4 (1)

Values are *n* (%). TTO, time trade-off; VAS, visual analogue scale; SG, standard gamble; C-SG, chained standard gamble; SF-6D, Short-Form 6D; HUI-2, Health Utilities Index-2; HUI-3, Health Utilities Index-3.

### Pooled utility estimates

Pooled utility estimates were obtained for sufficiently homogeneous health states. There was a sufficient number of studies reporting utility values in patients diagnosed with CTS and hand osteoarthritis who had not received any form of surgical intervention. To ensure homogeneity, meta-analysis was conducted using utility estimates obtained through indirect techniques. Collectively, 26 utility estimates for CTS were identified from seven studies and 22 utility estimates for hand osteoarthritis were identified from nine studies. Multiple HSUV estimates were obtained from individual studies. Where reported, these have been included separately in the meta-analysis. *Tables S1*, *S2* provide further descriptions of health states included in pooled utility estimates.

**Fig. 2:**
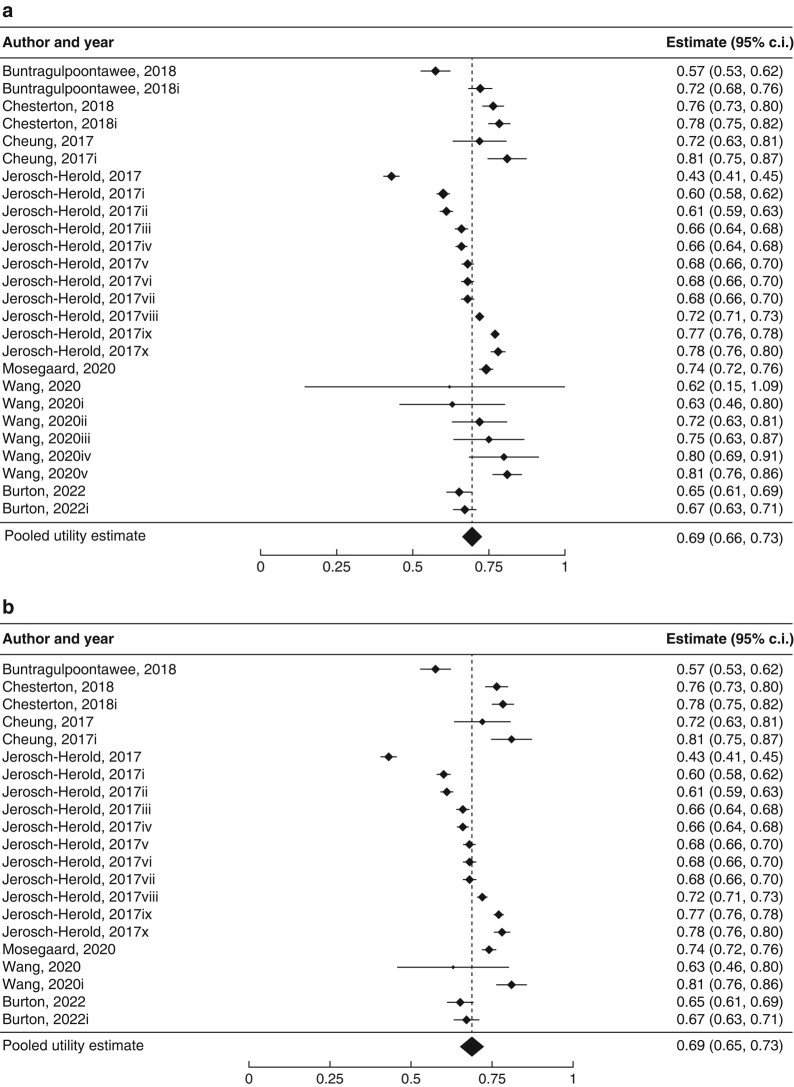
Forest plots demonstrating pooled utility estimates for baseline carpal tunnel syndrome (before surgical intervention) **a** Measured using all indirect instruments. **b** Measured using the EQ-5D only. Utility values from distinct cohorts in the same primary study are indicated. Please refer to *Table S2* for full descriptions of health states.

The pooled utility estimate for patients diagnosed with CTS who had not undergone surgical intervention across all studies using indirect valuation techniques was 0.69 (95% c.i. 0.66 to 0.73; *I*^2^ = 98%; 26 studies) (*Fig. 2a*). The pooled EQ-5D index estimate in patients with CTS who had not undergone surgical intervention was 0.69 (95% c.i. 0.65 to 0.73; *I*^2^ = 98%; 21 studies) (*Fig. 2b*).

**Fig. 3:**
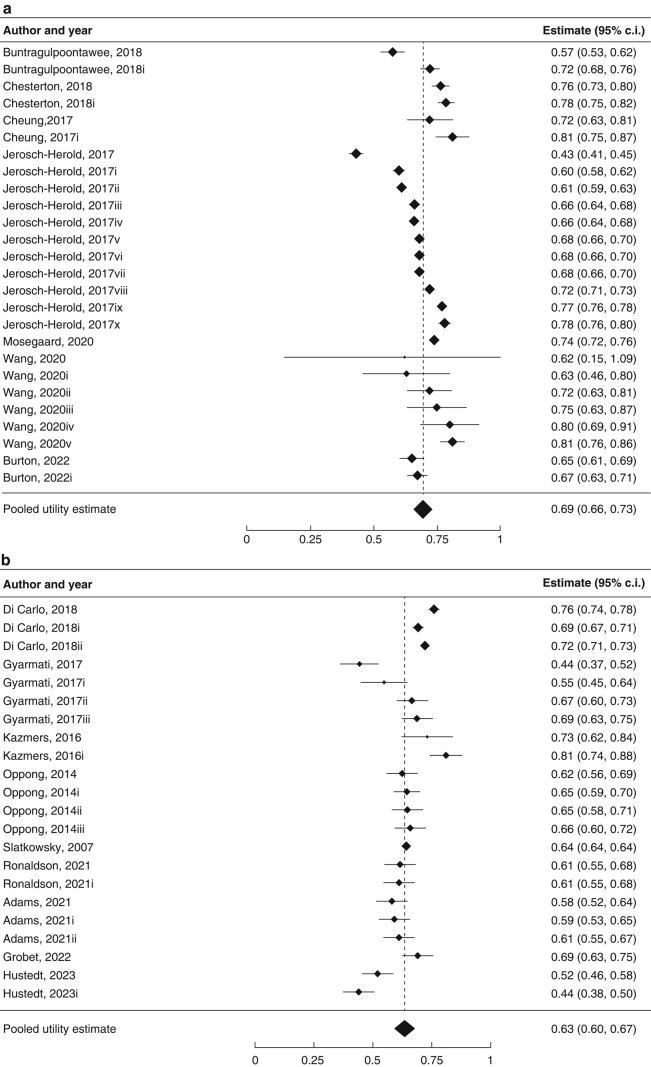
Forest plots demonstrating pooled utility estimates for baseline hand osteoarthritis (before surgical intervention) **a** Measured using all indirect instruments. **b** Measured using the EQ-5D only. Utility values from distinct cohorts in the same primary study are indicated. Please refer to *Table S2* for full descriptions of health states.

The pooled utility estimate for patients diagnosed with hand osteoarthritis who had not undergone surgical intervention across all studies using indirect valuation techniques was 0.63 (95% c.i. 0.60 to 0.67; *I*^2^ = 97%; 22 studies) (*Fig. 3a*). The pooled EQ-5D index estimate in patients with hand osteoarthritis who had not undergone surgical intervention was 0.64 (95% c.i. 0.60 to 0.68; *I*^2^ = 89%; 17 studies) (*Fig. 3b*).

## Discussion

This systematic review provides catalogued utility values for 363 health states experienced by patients with conditions affecting the hand. Pooled utility estimates were obtained for CTS and hand osteoarthritis before surgical intervention. The pooled EQ-5D utility score for CTS was 0.69. This is 11% lower than age-matched population norms and is equivalent to an individual who has severe problems with self-care and moderate problems carrying out everyday activities^22^. The pooled EQ-5D utility score for hand osteoarthritis was 0.64. This is 18% lower than age-matched population norms and is equivalent to an individual who has moderate difficulty walking, moderate problems carrying out daily activities, and moderate pain^22^. Pooled utility values for both CTS and hand osteoarthritis suggest substantial impairments in quality of life, particularly compared with utility values for chronic conditions, such as chronic obstructive pulmonary disease (pooled HSUV 0.71)^23^ and coronary artery disease (pooled HSUV 0.76)^24^.

This is the first study to catalogue all HSUVs experienced by patients with electively managed conditions of the hand. A recent study conducted a broad systematic review of HSUVs in the plastic surgery literature and only identified 13 studies that reported utility values for conditions affecting the hand and wrist. This systematic review provides catalogued utility values for 363 distinct health states for use in future economic analyses. This difference may be in part explained by the broader search strategy of the present review. Further, pooled HSUV estimates in this meta-analysis can be used by analysts to populate decision models and in cost-utility analyses of interventions for CTS and hand osteoarthritis. There was, however, a high degree of between-study heterogeneity observed and caution should be exercised.

Heterogeneity in HSUV estimates may, in part, be explained by differences in valuation technique, condition severity, and valuing population between individual utility derivation studies. The effect of valuation instrument on HSUVs is well established. Wang *et al*.^6^ conducted a retrospective cohort study in 29 patients diagnosed with CTS and compared HSUVs obtained through the SF-6D, the EQ-5D-5L, VAS, and CSG. Patients were stratified into mild/moderate and severe cohorts based on responses to the Boston Carpal Tunnel Questionnaire. In patients with mild/moderate CTS there were noted differences in median utility values derived using generic preference-based measures (PBMs): SF-6D 0.72 (i.q.r. 0.19) *versus* EQ-5D-5L 0.81 (i.q.r. 0.10) *versus* VAS 0.75 (i.q.r. 0.25). Interestingly, study participants were also asked to value their current health state through CSG. This technique employs an ‘anchor state’ in place of the risk of death, which is used in conventional SG. The use of an anchor state in CSG has been shown to have better interpretability^25^. Wang *et al*.^6^ chose median nerve transection as the anchor state in their CSG. When valuing their current health state using CSG, participants with mild/moderate CTS had a median utility of 0.97 (i.q.r. 0.03). The effect of valuation technique on utility value was also observed for patients with severe CTS. Median utility values obtained using indirect techniques were substantially lower than those obtained using CSG: SF-6D 0.62 (i.q.r. 0.80) *versus* EQ-5D-5L 0.63 (i.q.r. 0.29) *versus* VAS 0.80 (i.q.r. 0.19) *versus* CSG 0.99 (i.q.r. 0.01). SG has been considered the gold standard for utility derivation, as it incorporates risk and is underpinned by expected utility theory. However, probability weighting, loss aversion, and scale compatibility introduce bias and generally lead to SG utility values that are higher than expected^26^. Further, application of SG is limited due to high resource demands compared with indirect measures, such as the EQ-5D.

Conditions with known substantial quality-of-life implications, such as amputation, had higher HSUVs compared with seemingly innocuous conditions, such as CTS, in some publications. For example, Alolabi *et al*.^27^ report an HSUV of 0.72 for hand amputation (valued by members of the public), whereas Buntragulpoontawee *et al*.^28^ report an HSUV of 0.56 for baseline CTS. This difference may, in part, be explained by respondent heterogeneity. HSUVs derived from patients may be subject to perspective distortion, due to their experience living with the condition being valued, leading to a downward bias in utility estimates. This is generally why technology appraisal organizations, such as NICE, express a preference for QALYs derived using the EQ-5D, as its health states have been valued by members of the general population.

The majority of HSUVs identified in this systematic review were estimated using generic PBMs. Generic PBMs generate a health state classification system across multiple ‘generic’ domains of health and well-being. A preference-based scoring algorithm is derived from associated value sets and enables a utility value to be estimated for each described health state. The EQ-5D was the most used generic PBM in the present study. The use of generic PBMs has the obvious advantage of HSUV comparability across interventions. However, generic PBMs may not capture all elements of health that are important to patients with hand conditions. One solution to address the limitations of generic PBMs is the creation of specific preference-based measures. Such measures can be condition or domain specific (for example, specific to CTS or the hand as a whole). Specific PBMs generate a health state classification system across domains of health that are relevant to the patient cohort of interest. Specific PBMs have an associated value set that enables utilities to be estimated for the described health state. A number of specific PBMs have been described^29–31^; the present study did not identify any specific PBMs relevant to electively managed conditions of the hand. Specific PBMs can be derived *de novo* or they can be adapted from an existing PROM through the creation of a value set. In the creation of a specific PBM, members of the general population are asked to value states described by the PROM using either SG or TTO. Once a value set for the PROM has been generated, HSUVs can be determined and applied prospectively and retrospectively to existing data sets. The obvious limitations of specific PBMs are the limited comparability across conditions and the failure to capture systemic side effects of treatment.

The present study has potential limitations. The generalizability of pooled utility estimates is limited by high levels of between-study heterogeneity. This may, in part, be explained by differences in condition severity, prior treatment, valuation technique, and study population. Analysts populating decision models and conducting economic analyses of interventions should interpret pooled estimates with caution. Stratification by condition severity and treatment received was not planned due to anticipated paucity of data and lack of standardized severity assessment. A comparative analysis of utility values obtained through the EQ-5D-3L and the EQ-5D-5L was not conducted and index values were pooled instead due to a lack of primary data. The impact of covariates, such as country, valuation technique, and sex, on utility values was not evaluated through meta-regression due to the limited number of included studies and the high number of potential covariates^19^. Finally, the impact of selection bias in primary studies must be considered when interpreting utility estimates. Analysis of generic PBMs from observational studies and registry-based cohort studies may, in part, ensure that pooled HSUV estimates are representative of all patients, including those likely to be excluded from primary studies seeking to determine intervention effect.

Outcome assessment using high-quality measurement instruments is a central tenet in the provision of value-based care. The International Consortium for Health Outcomes Measurement (ICHOM) is a collaborative, multistakeholder, multinational group that aims to reach consensus on appropriate outcome measures for given conditions. Recently, the ICHOM standard outcome measure set for hand and wrist conditions was published^32^. Out of 90 identified outcome tools, 15 were selected to form the hand and wrist standard set. Of these 15 measures, only 1 preference-based measure was identified, the EQ-5D. Current health economic processes, such as the NICE Technology Appraisal, are reliant on generic measures, such as the EQ-5D. Future work should seek to develop value sets for hand-specific PROMs collected routinely as part of the standard outcome measure set for hand and wrist conditions. This may enable more precise estimates of HSUVs to be derived from existing and future data from national registries and clinical trials. To ensure comparability of QALYs across conditions, such initiatives should continue collecting generic PBMs, such as the EQ-5D. Future work can then assess whether QALYs derived using hand-specific or generic measures significantly differ.

## Supplementary Material

znae067_Supplementary_Data

## Data Availability

All data collected for this study are presented in the manuscript and associated *Supplementary material*. Details related to excluded studies are available on request.
