# Supplementary material for: Health-related quality of life in patients with conditions affecting the hand: meta-analysis
Source: Br J Surg. 2024 Apr 9;111(4):znae067. doi: 10.1093/bjs/znae067 (PMC11003527; doi:10.1093/bjs/znae067)
Supplement: znae067_Supplementary_Data [file znae067_supplementary_data.zip › Supplementary_material (3).docx]

| **Author** | **Year** | **Country** | **Study type** | **Condition** | **N patients** | **Health state** | **Valuation method** | **Respondent** | **Average utility value** | **Standard deviation** |
| --- | --- | --- | --- | --- | --- | --- | --- | --- | --- | --- |
| Alolabi | 2015 | Canada | Utility derivation | Hand amputation | 12 | Hand amputation | TTO | Patient | 0.69 |  |
| Alolabi | 2015 | Canada | Utility derivation | Hand amputation | 12 | Hand amputation | SG | Patient | 0.70 |  |
| Alolabi | 2015 | Canada | Utility derivation | Hand amputation | 30 | Hand amputation | TTO | Public | 0.72 |  |
| Alolabi | 2015 | Canada | Utility derivation | Hand amputation | 30 | Hand amputation with transplant | TTO | Public | 0.74 |  |
| Alolabi | 2015 | Canada | Utility derivation | Hand amputation | 30 | Hand amputation | SG | Public | 0.80 |  |
| Alolabi | 2015 | Canada | Utility derivation | Hand amputation | 30 | Hand amputation with transplant | SG | Public | 0.82 |  |
| Alolabi | 2015 | Canada | Utility derivation | Hand amputation | 12 | Hand amputation with transplant | TTO | Patient | 0.83 |  |
| Alolabi | 2015 | Canada | Utility derivation | Hand amputation | 12 | Hand amputation with transplant | SG | Patient | 0.86 |  |
| Cavaliere | 2010 | USA | Decision analysis | RA | 49 | Conservative management | TTO | Patient | 0.41 |  |
| Cavaliere | 2010 | USA | Decision analysis | RA | 49 | Total wrist fusion no complication | TTO | Patient | 0.51 |  |
| Cavaliere | 2010 | USA | Decision analysis | RA | 49 | Total wrist fusion major complication | TTO | Patient | 0.54 |  |
| Cavaliere | 2010 | USA | Decision analysis | RA | 49 | Total wrist fusion intermediate complication | TTO | Patient | 0.55 |  |
| Cavaliere | 2010 | USA | Decision analysis | RA | 109 | Conservative management | TTO | Healthcare Professionals | 0.55 |  |
| Cavaliere | 2010 | USA | Decision analysis | RA | 49 | Total wrist fusion minor complication | TTO | Patient | 0.59 |  |
| Cavaliere | 2010 | USA | Decision analysis | RA | 49 | Total wrist arthroplasty major complication: arthrodesis | TTO | Patient | 0.60 |  |
| Cavaliere | 2010 | USA | Decision analysis | RA | 49 | Total wrist arthroplasty major complication: revision arthroplasty | TTO | Patient | 0.63 |  |
| Cavaliere | 2010 | USA | Decision analysis | RA | 49 | Total wrist arthroplasty minor complication | TTO | Patient | 0.64 |  |
| Cavaliere | 2010 | USA | Decision analysis | RA | 49 | Total wrist arthroplasty no complication | TTO | Patient | 0.68 |  |
| Cavaliere | 2010 | USA | Decision analysis | RA | 49 | Total wrist arthroplasty intermediate complication | TTO | Patient | 0.70 |  |
| Cavaliere | 2010 | USA | Decision analysis | RA | 109 | Total wrist fusion no complication | TTO | Healthcare Professionals | 0.80 |  |
| Cavaliere | 2010 | USA | Decision analysis | RA | 109 | Total wrist fusion major complication | TTO | Healthcare Professionals | 0.81 |  |
| Cavaliere | 2010 | USA | Decision analysis | RA | 109 | Total wrist arthroplasty major complication: arthrodesis | TTO | Healthcare Professionals | 0.81 |  |
| Cavaliere | 2010 | USA | Decision analysis | RA | 109 | Total wrist fusion minor complication | TTO | Healthcare Professionals | 0.82 |  |
| Cavaliere | 2010 | USA | Decision analysis | RA | 109 | Total wrist arthroplasty major complication: revision arthroplasty | TTO | Healthcare Professionals | 0.82 |  |
| Cavaliere | 2010 | USA | Decision analysis | RA | 109 | Total wrist fusion intermediate complication | TTO | Healthcare Professionals | 0.84 |  |
| Cavaliere | 2010 | USA | Decision analysis | RA | 109 | Total wrist arthroplasty no complication | TTO | Healthcare Professionals | 0.85 |  |
| Cavaliere | 2010 | USA | Decision analysis | RA | 109 | Total wrist arthroplasty minor complication | TTO | Healthcare Professionals | 0.86 |  |
| Cavaliere | 2010 | USA | Decision analysis | RA | 109 | Total wrist arthroplasty intermediate complication | TTO | Healthcare Professionals | 0.87 |  |
| Chen | 2011 | USA | Decision analysis | DD | 50 | Successful open partial fasciectomy + complex regional pain syndrome | SG | Public | 0.97 |  |
| Chen | 2011 | USA | Decision analysis | DD | 50 | Failed open partial fasciectomy + failed revision fasciectomy | SG | Public | 0.98 |  |
| Chen | 2011 | USA | Decision analysis | DD | 50 | Failed collagenase injection + failed revision fasciectomy | SG | Public | 0.98 |  |
| Chen | 2011 | USA | Decision analysis | DD | 50 | Failed needle aponeurotomy + failed revision fasciectomy | SG | Public | 0.98 |  |
| Chen | 2011 | USA | Decision analysis | DD | 50 | Failed open partial fasciectomy + successful revision fasciectomy | SG | Public | 0.99 |  |
| Chen | 2011 | USA | Decision analysis | DD | 50 | No treatment | SG | Public | 0.99 |  |
| Chen | 2011 | USA | Decision analysis | DD | 50 | Successful open partial fasciectomy + nerve injury | SG | Public | 0.99 |  |
| Chen | 2011 | USA | Decision analysis | DD | 50 | Successful needle aponeurotomy + nerve injury | SG | Public | 0.99 |  |
| Chen | 2011 | USA | Decision analysis | DD | 50 | Successful open partial fasciectomy | SG | Public | 0.99 |  |
| Chen | 2011 | USA | Decision analysis | DD | 50 | Failed needle aponeurotomy + successful revision fasciectomy | SG | Public | 0.99 |  |
| Chen | 2011 | USA | Decision analysis | DD | 50 | Successful needle aponeurotomy | SG | Public | 0.99 |  |
| Chen | 2011 | USA | Decision analysis | DD | 50 | Successful collagenase injection | SG | Public | 0.99 |  |
| Chen | 2011 | USA | Decision analysis | DD | 50 | Failed collagenase injection + successful revision fasciectomy | SG | Public | 0.99 |  |
| Chung | 2010 | USA | Utility derivation | Hand amputation | 100 | Bilateral hand transplantation with major immunosuppression complication | TTO | Medical Student | 0.53 |  |
| Chung | 2010 | USA | Utility derivation | Hand amputation | 100 | Unilateral hand transplantation with major immunosuppression complication | TTO | Medical Student | 0.59 |  |
| Chung | 2010 | USA | Utility derivation | Hand amputation | 100 | Bilateral hand transplantation with transplant failure | TTO | Medical Student | 0.62 |  |
| Chung | 2010 | USA | Utility derivation | Hand amputation | 100 | Bilateral hand amputation with prosthesis | TTO | Medical Student | 0.63 |  |
| Chung | 2010 | USA | Utility derivation | Hand amputation | 100 | Unilateral hand transplantation with transplant failure | TTO | Medical Student | 0.73 |  |
| Chung | 2010 | USA | Utility derivation | Hand amputation | 100 | Bilateral hand transplantation with minor immunosuppression complication | TTO | Medical Student | 0.73 |  |
| Chung | 2010 | USA | Utility derivation | Hand amputation | 100 | Unilateral hand amputation with prosthesis | TTO | Medical Student | 0.75 |  |
| Chung | 2010 | USA | Utility derivation | Hand amputation | 100 | Unilateral hand transplantation with minor immunosuppression complication | TTO | Medical Student | 0.78 |  |
| Efanov | 2019 | Canada | Economic evaluation | Base of thumb osteoarthritis | 32 | Base of thumb osteoarthritis with severe pain | SG | Patient | 0.75 | 0.27 |
| Efanov | 2019 | Canada | Economic evaluation | Base of thumb osteoarthritis | 32 | Base of thumb osteoarthritis with severe pain | TTO | Patient | 0.84 | 0.15 |
| Efanov | 2022 | USA, Canada | Utility derivation | Hand amputation | 5 | Hand transplantation without complications | VAS | Patient - bilateral amputees | 0.92 | 0.08 |
| Efanov | 2022 | USA, Canada | Utility derivation | Hand amputation | 5 | Hand transplantation without complications | TTO | Patient - bilateral amputees | 0.80 | 0.05 |
| Efanov | 2022 | USA, Canada | Utility derivation | Hand amputation | 5 | Hand transplantation without complications | SG | Patient - bilateral amputees | 0.80 | 0.08 |
| Efanov | 2022 | USA, Canada | Utility derivation | Hand amputation | 5 | Myoelectric prosthesis without complications | VAS | Patient - bilateral amputees | 0.78 | 0.22 |
| Efanov | 2022 | USA, Canada | Utility derivation | Hand amputation | 5 | Myoelectric prosthesis without complications | TTO | Patient - bilateral amputees | 0.71 | 0.16 |
| Efanov | 2022 | USA, Canada | Utility derivation | Hand amputation | 5 | Myoelectric prosthesis without complications | SG | Patient - bilateral amputees | 0.68 | 0.11 |
| Efanov | 2022 | USA, Canada | Utility derivation | Hand amputation | 5 | Hand transplantation with significant complications | VAS | Patient - bilateral amputees | 0.56 | 0.20 |
| Efanov | 2022 | USA, Canada | Utility derivation | Hand amputation | 5 | Hand transplantation with significant complications | TTO | Patient - bilateral amputees | 0.58 | 0.08 |
| Efanov | 2022 | USA, Canada | Utility derivation | Hand amputation | 5 | Hand transplantation with significant complications | SG | Patient - bilateral amputees | 0.51 | 0.04 |
| Efanov | 2022 | USA, Canada | Utility derivation | Hand amputation | 5 | Myoelectric prosthesis with significant complications | VAS | Patient - bilateral amputees | 0.61 | 0.18 |
| Efanov | 2022 | USA, Canada | Utility derivation | Hand amputation | 5 | Myoelectric prosthesis with significant complications | TTO | Patient - bilateral amputees | 0.52 | 0.14 |
| Efanov | 2022 | USA, Canada | Utility derivation | Hand amputation | 5 | Myoelectric prosthesis with significant complications | SG | Patient - bilateral amputees | 0.50 | 0.13 |
| Efanov | 2022 | USA, Canada | Utility derivation | Hand amputation | 12 | Hand transplantation without complications | VAS | Patient - bilateral amputees | 0.87 | 0.11 |
| Efanov | 2022 | USA, Canada | Utility derivation | Hand amputation | 12 | Hand transplantation without complications | TTO | Patient - bilateral amputees | 0.83 | 0.11 |
| Efanov | 2022 | USA, Canada | Utility derivation | Hand amputation | 12 | Hand transplantation without complications | SG | Patient - bilateral amputees | 0.84 | 0.06 |
| Efanov | 2022 | USA, Canada | Utility derivation | Hand amputation | 12 | Myoelectric prosthesis without complications | VAS | Patient - bilateral amputees | 0.93 | 0.09 |
| Efanov | 2022 | USA, Canada | Utility derivation | Hand amputation | 12 | Myoelectric prosthesis without complications | TTO | Patient - bilateral amputees | 0.84 | 0.05 |
| Efanov | 2022 | USA, Canada | Utility derivation | Hand amputation | 12 | Myoelectric prosthesis without complications | SG | Patient - bilateral amputees | 0.81 | 0.05 |
| Efanov | 2022 | USA, Canada | Utility derivation | Hand amputation | 12 | Hand transplantation with significant complications | VAS | Patient - bilateral amputees | 0.45 | 0.12 |
| Efanov | 2022 | USA, Canada | Utility derivation | Hand amputation | 12 | Hand transplantation with significant complications | TTO | Patient - bilateral amputees | 0.29 | 0.13 |
| Efanov | 2022 | USA, Canada | Utility derivation | Hand amputation | 12 | Hand transplantation with significant complications | SG | Patient - bilateral amputees | 0.30 | 0.11 |
| Efanov | 2022 | USA, Canada | Utility derivation | Hand amputation | 12 | Myoelectric prosthesis with significant complications | VAS | Patient - bilateral amputees | 0.55 | 0.14 |
| Efanov | 2022 | USA, Canada | Utility derivation | Hand amputation | 12 | Myoelectric prosthesis with significant complications | TTO | Patient - bilateral amputees | 0.45 | 0.08 |
| Efanov | 2022 | USA, Canada | Utility derivation | Hand amputation | 12 | Myoelectric prosthesis with significant complications | SG | Patient - bilateral amputees | 0.42 | 0.06 |
| Efanov | 2022 | USA, Canada | Utility derivation | Hand amputation | 9 | Hand transplantation without complications | VAS | Patient - proximal replantation | 0.80 | 0.14 |
| Efanov | 2022 | USA, Canada | Utility derivation | Hand amputation | 9 | Hand transplantation without complications | TTO | Patient - proximal replantation | 0.77 | 0.04 |
| Efanov | 2022 | USA, Canada | Utility derivation | Hand amputation | 9 | Hand transplantation without complications | SG | Patient - proximal replantation | 0.79 | 0.06 |
| Efanov | 2022 | USA, Canada | Utility derivation | Hand amputation | 9 | Myoelectric prosthesis without complications | VAS | Patient - proximal replantation | 0.90 | 0.05 |
| Efanov | 2022 | USA, Canada | Utility derivation | Hand amputation | 9 | Myoelectric prosthesis without complications | TTO | Patient - proximal replantation | 0.88 | 0.07 |
| Efanov | 2022 | USA, Canada | Utility derivation | Hand amputation | 9 | Myoelectric prosthesis without complications | SG | Patient - proximal replantation | 0.83 | 0.05 |
| Efanov | 2022 | USA, Canada | Utility derivation | Hand amputation | 9 | Hand transplantation with significant complications | VAS | Patient - proximal replantation | 0.44 | 0.15 |
| Efanov | 2022 | USA, Canada | Utility derivation | Hand amputation | 9 | Hand transplantation with significant complications | TTO | Patient - proximal replantation | 0.34 | 0.11 |
| Efanov | 2022 | USA, Canada | Utility derivation | Hand amputation | 9 | Hand transplantation with significant complications | SG | Patient - proximal replantation | 0.32 | 0.10 |
| Efanov | 2022 | USA, Canada | Utility derivation | Hand amputation | 9 | Myoelectric prosthesis with significant complications | VAS | Patient - proximal replantation | 0.68 | 0.19 |
| Efanov | 2022 | USA, Canada | Utility derivation | Hand amputation | 9 | Myoelectric prosthesis with significant complications | TTO | Patient - proximal replantation | 0.55 | 0.05 |
| Efanov | 2022 | USA, Canada | Utility derivation | Hand amputation | 9 | Myoelectric prosthesis with significant complications | SG | Patient - proximal replantation | 0.53 | 0.08 |
| Efanov | 2022 | USA, Canada | Utility derivation | Hand amputation | 45 | Hand transplantation without complications | VAS | Public | 0.65 | 0.18 |
| Efanov | 2022 | USA, Canada | Utility derivation | Hand amputation | 45 | Hand transplantation without complications | TTO | Public | 0.63 | 0.15 |
| Efanov | 2022 | USA, Canada | Utility derivation | Hand amputation | 45 | Hand transplantation without complications | SG | Public | 0.60 | 0.08 |
| Efanov | 2022 | USA, Canada | Utility derivation | Hand amputation | 45 | Myoelectric prosthesis without complications | VAS | Public | 0.62 | 0.23 |
| Efanov | 2022 | USA, Canada | Utility derivation | Hand amputation | 45 | Myoelectric prosthesis without complications | TTO | Public | 0.52 | 0.06 |
| Efanov | 2022 | USA, Canada | Utility derivation | Hand amputation | 45 | Myoelectric prosthesis without complications | SG | Public | 0.51 | 0.07 |
| Efanov | 2022 | USA, Canada | Utility derivation | Hand amputation | 45 | Hand transplantation with significant complications | VAS | Public | 0.40 | 0.14 |
| Efanov | 2022 | USA, Canada | Utility derivation | Hand amputation | 45 | Hand transplantation with significant complications | TTO | Public | 0.26 | 0.09 |
| Efanov | 2022 | USA, Canada | Utility derivation | Hand amputation | 45 | Hand transplantation with significant complications | SG | Public | 0.24 | 0.04 |
| Efanov | 2022 | USA, Canada | Utility derivation | Hand amputation | 45 | Myoelectric prosthesis with significant complications | VAS | Public | 0.46 | 0.17 |
| Efanov | 2022 | USA, Canada | Utility derivation | Hand amputation | 45 | Myoelectric prosthesis with significant complications | TTO | Public | 0.27 | 0.07 |
| Efanov | 2022 | USA, Canada | Utility derivation | Hand amputation | 45 | Myoelectric prosthesis with significant complications | SG | Public | 0.24 | 0.05 |
| Harijee | 2020 | India | Economic evaluation | Hand amputation | 23 | Bilateral hand amputation | TTO | Patient (Bilateral Hand Amputees) | 0.32 | 0.25 |
| Harijee | 2020 | India | Economic evaluation | Hand amputation | 120 | Bilateral hand amputation | TTO | Healthcare Professionals | 0.34 | 0.22 |
| Harijee | 2020 | India | Economic evaluation | Hand amputation | 89 | Bilateral hand amputation | TTO | Public | 0.35 | 0.27 |
| Harijee | 2020 | India | Economic evaluation | Hand amputation | 23 | Bilateral hand amputation with prosthesis | TTO | Patient (Bilateral Hand Amputees) | 0.38 | 0.28 |
| Harijee | 2020 | India | Economic evaluation | Hand amputation | 89 | Bilateral hand amputation with prosthesis | TTO | Public | 0.50 | 0.26 |
| Harijee | 2020 | India | Economic evaluation | Hand amputation | 120 | Bilateral hand amputation with prosthesis | TTO | Healthcare Professionals | 0.52 | 0.24 |
| Harijee | 2020 | India | Economic evaluation | Hand amputation | 120 | Bilateral hand amputation with transplant | TTO | Healthcare Professionals | 0.63 | 0.25 |
| Harijee | 2020 | India | Economic evaluation | Hand amputation | 23 | Bilateral hand amputation with transplant | TTO | Patient (Bilateral Hand Amputees) | 0.74 | 0.24 |
| Harijee | 2020 | India | Economic evaluation | Hand amputation | 89 | Bilateral hand amputation with transplant | TTO | Public | 0.74 | 0.25 |
| Harijee | 2021 | India | Cross-sectional study | Bilateral hand amputation | 32 | Hand amputation | TTO | Patient | 0.34 | 0.25 |
| McCabe | 1998 | USA | RCT | Upper extremity amputatuon | 22 | Upper extremity amputatuon; success of transplant with poor health | TTO | Public | 0.64 |  |
| McCabe | 1998 | USA | RCT | Upper extremity amputatuon | 22 | Upper extremity amputatuon; transplant loss with good health | TTO | Public | 0.90 |  |
| McCabe | 1998 | USA | RCT | Upper extremity amputatuon | 22 | Upper extremity amputatuon; no transplant | TTO | Public | 0.93 |  |
| McCabe | 1998 | USA | RCT | Upper extremity amputatuon | 22 | Upper extremity amputatuon; success of transplant with good health | TTO | Public | 0.96 |  |
| Tessler | 2017 | Canada | Retrospective cohort | Digital replant | 36 | Digital replant | SG | Patient | 0.84 |  |
| Tessler | 2017 | Canada | Retrospective cohort | Digital amputation | 15 | Digital amputation | SG | Patient | 0.86 |  |
| Tessler | 2017 | Canada | Retrospective cohort | Digital replant | 36 | Digital replant | TTO | Patient | 0.86 |  |
| Tessler | 2017 | Canada | Retrospective cohort | Digital amputation | 15 | Digital amputation | TTO | Patient | 0.88 |  |
| Wang | 2020 | Canada | Economic evaluation | CTS | 29 | Treated CTS with median nerve transection | Chained SG | Patient | 0.95 |  |
| Wang | 2020 | Canada | Economic evaluation | CTS | 18 | Mild to moderate CTS | Chained SG | Patient | 0.97 | 0.03 |
| Wang | 2020 | Canada | Economic evaluation | CTS | 11 | Severe CTS | Chained SG | Patient | 0.99 | 0.01 |
| CTS – Carpal Tunnel Syndrome; CRPS – Complex Regional Pain Syndrome; DD – Dupuytren’s Disease; RA – Rheumatoid Arthritis; RCT – Randomised Control Trial; SG – Standard Gamble; TTO – Time Trade Off | | | | | | | | | |  |

**Supplementary Table 1:** Catalogued average utility values derived using direct valuation techniques with associated health states. Mean utility values are presented unless stated otherwise.

| **Author** | **Year** | **Country** | **Study type** | **Condition** | **N patients** | **Health state** | **Valuation method** | **Respondent** | | **Value set (if applicable)** | **Average utility value** | **Standard deviation** |
| --- | --- | --- | --- | --- | --- | --- | --- | --- | --- | --- | --- | --- |
| Adams | 2021 | UK | RCT | OA | 116 | Symptomatic base of thumb osteoarthritis - baseline | EQ-5D-5L | Patient | | UK | 0.58 | 0.23 |
| Adams | 2021 | UK | RCT | OA | 116 | Symptomatic base of thumb osteoarthritis - therapy (2 months) | EQ-5D-5L | Patient | | UK | 0.65 | 0.21 |
| Adams | 2021 | UK | RCT | OA | 116 | Symptomatic base of thumb osteoarthritis - therapy (3 months) | EQ-5D-5L | Patient | | UK | 0.61 | 0.24 |
| Adams | 2021 | UK | RCT | OA | 116 | Symptomatic base of thumb osteoarthritis - baseline | EQ-5D-5L | Patient | | UK | 0.59 | 0.21 |
| Adams | 2021 | UK | RCT | OA | 116 | Symptomatic base of thumb osteoarthritis - therapy + splint (2 months) | EQ-5D-5L | Patient | | UK | 0.65 | 0.17 |
| Adams | 2021 | UK | RCT | OA | 116 | Symptomatic base of thumb osteoarthritis - therapy + splint (3 months) | EQ-5D-5L | Patient | | UK | 0.63 | 0.22 |
| Adams | 2021 | UK | RCT | OA | 117 | Symptomatic base of thumb osteoarthritis - baseline | EQ-5D-5L | Patient | | UK | 0.61 | 0.21 |
| Adams | 2021 | UK | RCT | OA | 117 | Symptomatic base of thumb osteoarthritis - therapy + placebo splint (2 months) | EQ-5D-5L | Patient | | UK | 0.63 | 0.2 |
| Adams | 2021 | UK | RCT | OA | 117 | Symptomatic base of thumb osteoarthritis - therapy + placebo splint (3 months) | EQ-5D-5L | Patient | | UK | 0.64 | 0.21 |
| Atroshi | 2007 | USA | Prospective cohort | CTS | 100 | Baseline | SF-6D | Patient | | UK | 0.69 | 0.13 |
| Atroshi | 2007 | USA | Prospective cohort | CTS | 95 | 3 months post endoscopic release | SF-6D | Patient | | UK | 0.77 | 0.13 |
| Bjurehed | 2007 | Sweden | Prospective cohort | OA | 49 | Baseline | VAS | Patient | |  | 0.75* | - |
| Bjurehed | 2007 | Sweden | Prospective cohort | OA | 49 | 3 months after intervention (physiotherapy, education) | VAS | Patient | |  | 0.80* | - |
| Bjurehed | 2007 | Sweden | Prospective cohort | OA | 49 | 12 months after intervention (physiotherapy, education) | VAS | Patient | |  | 0.80* | - |
| Buntragulpoontawee | 2018 | Thailand | Prospective cohort | CTS | 50 | Baseline | EQ-5D | Patient | | Thai | 0.56 | 0.17 |
| Buntragulpoontawee | 2018 | Thailand | Prospective cohort | CTS | 50 | Baseline | VAS | Patient | |  | 0.72 | 0.14 |
| Burton | 2022 | UK | RCT | CTS | 116 | Mild-to-moderate CTS - baseline | EQ‐5D‐5L | Patient | | UK | 0·652 | 0·22 |
| Burton | 2022 | UK | RCT | CTS | 116 | Mild-to-moderate CTS - steroid (6 weeks) | EQ‐5D‐5L | Patient | | UK | 0·703 | 0·23 |
| Burton | 2022 | UK | RCT | CTS | 116 | Mild-to-moderate CTS - steroid (6 months) | EQ‐5D‐5L | Patient | | UK | 0·732 | 0·18 |
| Burton | 2022 | UK | RCT | CTS | 116 | Mild-to-moderate CTS - steroid (12 months) | EQ‐5D‐5L | Patient | | UK | 0·742 | 0·19 |
| Burton | 2022 | UK | RCT | CTS | 116 | Mild-to-moderate CTS - steroid (24 months) | EQ‐5D‐5L | Patient | | UK | 0·733 | 0·18 |
| Burton | 2022 | UK | RCT | CTS | 118 | Mild-to-moderate CTS - baseline | EQ‐5D‐5L | Patient | | UK | 0·671 | 0·21 |
| Burton | 2022 | UK | RCT | CTS | 118 | Mild-to-moderate CTS - resting night splint (6 weeks) | EQ‐5D‐5L | Patient | | UK | 0·695 | 0·20 |
| Burton | 2022 | UK | RCT | CTS | 118 | Mild-to-moderate CTS - resting night splint (6 months) | EQ‐5D‐5L | Patient | | UK | 0·749 | 0·19 |
| Burton | 2022 | UK | RCT | CTS | 118 | Mild-to-moderate CTS - resting night splint (12 months) | EQ‐5D‐5L | Patient | | UK | 0·768 | 0·17 |
| Burton | 2022 | UK | RCT | CTS | 118 | Mild-to-moderate CTS - resting night splint (24 months) | EQ‐5D‐5L | Patient | | UK | 0·755 | 0·20 |
| Caplan | 2017 | UK | Prospective cohort | Hand and wrist pain | 84 | Baseline | EQ-5D | Patient | | UK | 0.70 | 0.22 |
| Caplan | 2017 | UK | Prospective cohort | Hand and wrist pain | 84 | Hand and wrist pain after community physiotherapy | EQ-5D | Patient | | UK | 0.86 | 0.14 |
| Chesterton | 2018 | UK | RCT | CTS | 116 | Baseline (cohort 1) | EQ-5D | Patient | | UK | 0.76 | 0.19 |
| Chesterton | 2018 | UK | RCT | CTS | 118 | Night splint 6 weeks | EQ-5D | Patient | | UK | 0.78 | 0.21 |
| Chesterton | 2018 | UK | RCT | CTS | 118 | Baseline (cohort 2) | EQ-5D | Patient | | UK | 0.78 | 0.19 |
| Chesterton | 2018 | UK | RCT | CTS | 116 | Steroid 6 weeks | EQ-5D | Patient | | UK | 0.79 | 0.20 |
| Chesterton | 2018 | UK | RCT | CTS | 116 | Steroid 6 months | EQ-5D | Patient | | UK | 0.82 | 0.16 |
| Chesterton | 2018 | UK | RCT | CTS | 118 | Night splint 6 months | EQ-5D | Patient | | UK | 0.83 | 0.17 |
| Cheung | 2017 | Canada | Prospective cohort | CTS | 21 | Baseline | EQ-5D | Patient | | USA | 0.72 | 0.20 |
| Cheung | 2017 | USA | Prospective cohort | CTS | 8 | Baseline | EQ-5D | Patient | | USA | 0.81 | 0.09 |
| Cheung | 2017 | Canada | Prospective cohort | CTS | 21 | Open release 3 months | EQ-5D | Patient | | USA | 0.83 | 0.16 |
| Cheung | 2017 | USA | Prospective cohort | CTS | 8 | Open release 3 months | EQ-5D | Patient | | USA | 0.86 | 0.12 |
| Chung | 1998 | USA | Economic analysis | CTS | 12 | Transection of median nerve | VAS | Physician | |  | 0.48 | 0.19 |
| Chung | 1998 | USA | Economic analysis | CTS | 11 | Transection of median nerve | VAS | Nurse | |  | 0.55 | 0.16 |
| Chung | 1998 | USA | Economic analysis | CTS | 12 | Persistent symptoms for rest of life | VAS | Physician | |  | 0.65 | 0.19 |
| Chung | 1998 | USA | Economic analysis | CTS | 12 | Scar tenderness for 6 months | VAS | Physician | |  | 0.69 | 0.18 |
| Chung | 1998 | USA | Economic analysis | CTS | 11 | Scar tenderness for 6 months | VAS | Nurse | |  | 0.69 | 0.16 |
| Chung | 1998 | USA | Economic analysis | CTS | 12 | Wound infection for 3 weeks | VAS | Physician | |  | 0.75 | 0.20 |
| Chung | 1998 | USA | Economic analysis | CTS | 11 | Persistent symptoms for rest of life | VAS | Nurse | |  | 0.75 | 0.11 |
| Chung | 1998 | USA | Economic analysis | CTS | 11 | Wound infection for 3 weeks | VAS | Nurse | |  | 0.76 | 0.16 |
| Chung | 1998 | USA | Economic analysis | CTS | 11 | Open release | VAS | Nurse | |  | 0.76 | 0.15 |
| Chung | 1998 | USA | Economic analysis | CTS | 12 | Open release | VAS | Physician | |  | 0.80 | 0.14 |
| Chung | 1998 | USA | Economic analysis | CTS | 12 | Finger numbness for 3 months | VAS | Physician | |  | 0.81 | 0.12 |
| Chung | 1998 | USA | Economic analysis | CTS | 11 | Finger numbness for 3 months | VAS | Nurse | |  | 0.82 | 0.12 |
| Chung | 1998 | USA | Economic analysis | CTS | 11 | Endoscopic release | VAS | Nurse | |  | 0.82 | 0.13 |
| Chung | 1998 | USA | Economic analysis | CTS | 12 | Endoscopic release | VAS | Physician | |  | 0.83 | 0.11 |
| Coen | 2020 | Netherlands | Retrospective cohort | Various hand/wrist conditions | 616 | Various conditions during COVID-19 lockdown | EQ-5D | Patient | | Netherlands | 0.85 | - |
| Coen | 2020 | Netherlands | Retrospective cohort | Various hand/wrist conditions | 997 | Various conditions before COVID-19 lockdown | EQ-5D | Patient | | Netherlands | 0.85 | - |
| Coen | 2020 | Netherlands | Retrospective cohort | Various hand/wrist conditions | 997 | Various conditions before COVID-19 lockdown | VAS | Patient | | Netherlands | 0.85 | - |
| Coen | 2020 | Netherlands | Retrospective cohort | Various hand/wrist conditions | 616 | Various conditions during COVID-19 lockdown | VAS | Patient | | Netherlands | 0.86 | - |
| Dhawan | 2019 | China | Retrospective cohort | DD | 35 | Baseline | EQ-5D | Patient | | Unspecified | 0.79 | 0.26 |
| Dhawan | 2019 | China | Retrospective cohort | DD | 35 | 24 months after collagenase | EQ-5D | Patient | | Unspecified | 0.89 | 0.40 |
| Di Carlo | 2018 | Italy | Prospective cohort | OA | 87 | Baseline | EQ-5D | Patient | | Italy | 0.76 | 0.10 |
| Di Carlo | 2019 | Italy | Prospective cohort | OA | 87 | Baseline | SF-6D | Patient | | Italy | 0.69 | 0.10 |
| Di Carlo | 2020 | Italy | Prospective cohort | OA | 87 | Baseline | VAS | Patient | | Italy | 0.72 | 0.06 |
| Dritsaki | 2017 | UK | RCT | RA | 113 | Rheumatoid arthritis worsened following treatment 4 months | EQ-5D | Patient | | UK | 0.48 | - |
| Dritsaki | 2017 | UK | RCT | RA | 113 | Rheumatoid arthritis worsened following treatment baseline | EQ-5D | Patient | | UK | 0.55 | - |
| Dritsaki | 2017 | UK | RCT | RA | 113 | Rheumatoid arthritis worsened following treatment 12 months | EQ-5D | Patient | | UK | 0.57 | - |
| Dritsaki | 2017 | UK | RCT | RA | 177 | Rheumatoid arthritis improved following treatment baseline | EQ-5D | Patient | | UK | 0.58 | - |
| Dritsaki | 2017 | UK | RCT | RA | 113 | Rheumatoid arthritis worsened following treatment 4 months | SF-6D | Patient | |  | 0.59 | - |
| Dritsaki | 2017 | UK | RCT | RA | 113 | Rheumatoid arthritis worsened following treatment 12 months | VAS | Patient | |  | 0.59 | - |
| Dritsaki | 2017 | UK | RCT | RA | 113 | Rheumatoid arthritis worsened following treatment baseline | SF-6D | Patient | |  | 0.60 | - |
| Dritsaki | 2017 | UK | RCT | RA | 113 | Rheumatoid arthritis worsened following treatment 4 months | VAS | Patient | |  | 0.60 | - |
| Dritsaki | 2017 | UK | RCT | RA | 113 | Rheumatoid arthritis worsened following treatment 12 months | SF-6D | Patient | |  | 0.60 | - |
| Dritsaki | 2017 | UK | RCT | RA | 150 | Rheumatoid arthritis no change following treatment baseline | EQ-5D | Patient | | UK | 0.61 | - |
| Dritsaki | 2017 | UK | RCT | RA | 150 | Rheumatoid arthritis no change following treatment 4 months | EQ-5D | Patient | | UK | 0.62 | - |
| Dritsaki | 2017 | UK | RCT | RA | 150 | Rheumatoid arthritis no change following treatment 12 months | EQ-5D | Patient | | UK | 0.64 | - |
| Dritsaki | 2017 | UK | RCT | RA | 177 | Rheumatoid arthritis improved following treatment 12 months | SF-6D | Patient | |  | 0.64 | - |
| Dritsaki | 2017 | UK | RCT | RA | 113 | Rheumatoid arthritis worsened following treatment baseline | VAS | Patient | |  | 0.65 | - |
| Dritsaki | 2017 | UK | RCT | RA | 177 | Rheumatoid arthritis improved following treatment baseline | SF-6D | Patient | |  | 0.65 | - |
| Dritsaki | 2017 | UK | RCT | RA | 150 | Rheumatoid arthritis no change following treatment baseline | SF-6D | Patient | |  | 0.65 | - |
| Dritsaki | 2017 | UK | RCT | RA | 150 | Rheumatoid arthritis no change following treatment 4 months | SF-6D | Patient | |  | 0.65 | - |
| Dritsaki | 2017 | UK | RCT | RA | 150 | Rheumatoid arthritis no change following treatment 4 months | VAS | Patient | |  | 0.67 | - |
| Dritsaki | 2017 | UK | RCT | RA | 177 | Rheumatoid arthritis improved following treatment 12 months | EQ-5D | Patient | | UK | 0.67 | - |
| Dritsaki | 2017 | UK | RCT | RA | 150 | Rheumatoid arthritis no change following treatment 12 months | SF-6D | Patient | |  | 0.67 | - |
| Dritsaki | 2017 | UK | RCT | RA | 177 | Rheumatoid arthritis improved following treatment baseline | VAS | Patient | |  | 0.68 | - |
| Dritsaki | 2017 | UK | RCT | RA | 177 | Rheumatoid arthritis improved following treatment 4 months | SF-6D | Patient | |  | 0.68 | - |
| Dritsaki | 2017 | UK | RCT | RA | 150 | Rheumatoid arthritis no change following treatment baseline | VAS | Patient | |  | 0.69 | - |
| Dritsaki | 2017 | UK | RCT | RA | 177 | Rheumatoid arthritis improved following treatment 4 months | EQ-5D | Patient | | UK | 0.69 | - |
| Dritsaki | 2017 | UK | RCT | RA | 177 | Rheumatoid arthritis improved following treatment 12 months | EQ-5D | Patient | |  | 0.69 | - |
| Dritsaki | 2017 | UK | RCT | RA | 150 | Rheumatoid arthritis no change following treatment 12 months | EQ-5D | Patient | |  | 0.69 | - |
| Dritsaki | 2017 | UK | RCT | RA | 177 | Rheumatoid arthritis improved following treatment 4 months | EQ-5D | Patient | |  | 0.76 | - |
| Efanov | 2019 | Canada | Economic evaluation | Base of thumb OA | 32 | Base of thumb osteoarthritis with severe pain baseline | VAS | Patient | |  | 0.27 | 0.19 |
| Efanov | 2019 | Canada | Economic evaluation | Base of thumb OA | 32 | Base of thumb osteoarthritis with severe pain following trapeziectomy with LRTI | VAS | Patient | |  | 0.89 | 0.09 |
| Engstrand | 2014 | Sweden | Prospective cohort | DD | 81 | 3 months after fasciectomy + hand therapy | VAS | Patient | | Sweden | 0.79 | 0.17 |
| Engstrand | 2014 | Sweden | Prospective cohort | DD | 81 | Baseline | VAS | Patient | | Sweden | 0.80 | 0.15 |
| Engstrand | 2014 | Sweden | Prospective cohort | DD | 81 | Baseline | EQ-5D | Patient | | Sweden | 0.82 | 0.13 |
| Engstrand | 2014 | Sweden | Prospective cohort | DD | 81 | 3 months after fasciectomy + hand therapy | EQ-5D | Patient | | Sweden | 0.86 | 0.17 |
| Fernàndez-de-las-Peñas | 2018 | Spain | Economic evaluation | CTS | 120 | Physiotherapy | EQ-5D | Blinded examiner |  | | 0.61 | 0.09 |
| Fernàndez-de-las-Peñas | 2018 | Spain | Economic evaluation | CTS | 120 | Open decompression | EQ-5D | Blinded examiner |  | | 0.66 | 0.05 |
| Glinatsi | 2020 | Denmark | Observational | RA | 114 | Baseline RA treated with DMARDS | EQ-5D | Patient | |  | 0.87 | - |
| Glinatsi | 2020 | Denmark | Observational | RA | 70 | Relapsed RA treated with DMARDS | EQ-5D | Patient | |  | 0.87 | - |
| Grobet | 2022 | Switzerland | Prospective cohort | OA | 151 | Base of thumb osteoarthritis at enrollment | EQ‐5D‐5L | Patient | |  | 0.69 | 0.19 |
| Grobet | 2022 | Switzerland | Prospective cohort | OA | 151 | Base of thumb osteoarthritis pre-operative | EQ‐5D‐5L | Patient | |  | 0.69 | - |
| Grobet | 2022 | Switzerland | Prospective cohort | OA | 151 | Base of thumb osteoarthritis following resection arthroplasty (2 weeks) | EQ‐5D‐5L | Patient | |  | 0.75 | - |
| Grobet | 2022 | Switzerland | Prospective cohort | OA | 151 | Base of thumb osteoarthritis following resection arthroplasty (3 months) | EQ‐5D‐5L | Patient | |  | 0.85 | - |
| Grobet | 2022 | Switzerland | Prospective cohort | OA | 151 | Base of thumb osteoarthritis following resection arthroplasty (6 months) | EQ‐5D‐5L | Patient | |  | 0.86 | - |
| Grobet | 2022 | Switzerland | Prospective cohort | OA | 151 | Base of thumb osteoarthritis following resection arthroplasty (12 months) | EQ‐5D‐5L | Patient | |  | 0.88 | 0.11 |
| Gu | 2013 | UK | Discrete choice experiment | DD | 1745 | Completely affected dominant hand Dupuytren’s disease | EQ-5D | Patient | |  | 0.49 | - |
| Gu | 2013 | UK | Discrete choice experiment | DD | 1745 | Completely affected non dominant hand Dupuytren’s disease | EQ-5D | Patient | |  | 0.57 | - |
| Gu | 2013 | UK | Discrete choice experiment | DD | 1745 | Completely affected ambidexterous hand Dupuytren’s disease | EQ-5D | Patient | |  | 0.63 | - |
| Gyarmati | 2017 | Hungary | RCT | Mild to moderate OA | 24 | Hévíz mud to both hands with gloves, baseline | VAS | Patient | |  | 0.44 | 0.19 |
| Gyarmati | 2017 | Hungary | RCT | Mild to moderate OA | 23 | Hévíz mud applied directly to both hands, baseline | VAS | Patient | |  | 0.55 | 0.23 |
| Gyarmati | 2017 | Hungary | RCT | Mild to moderate OA | 24 | Hévíz mud to both hands with gloves, 4 months | VAS | Patient | |  | 0.58 | 0.22 |
| Gyarmati | 2017 | Hungary | RCT | Mild to moderate OA | 23 | Hévíz mud applied directly to both hands, 3 weeks | VAS | Patient | |  | 0.58 | 0.16 |
| Gyarmati | 2017 | Hungary | RCT | Mild to moderate OA | 24 | Hévíz mud to both hands with gloves, 3 weeks | VAS | Patient | |  | 0.59 | 0.21 |
| Gyarmati | 2017 | Hungary | RCT | Mild to moderate OA | 23 | Hévíz mud applied directly to both hands, 4 months | VAS | Patient | |  | 0.63 | 0.18 |
| Gyarmati | 2017 | Hungary | RCT | Mild to moderate OA | 24 | Hévíz mud to both hands with gloves, baseline | EQ-5D | Patient | |  | 0.67 | 0.15 |
| Gyarmati | 2017 | Hungary | RCT | Mild to moderate OA | 23 | Hévíz mud applied directly to both hands, baseline | EQ-5D | Patient | |  | 0.69 | 0.15 |
| Gyarmati | 2017 | Hungary | RCT | Mild to moderate OA | 24 | Hévíz mud to both hands with gloves, 3 weeks | EQ-5D | Patient | |  | 0.71 | 0.14 |
| Gyarmati | 2017 | Hungary | RCT | Mild to moderate OA | 24 | Hévíz mud to both hands with gloves, 4 months | EQ-5D | Patient | |  | 0.72 | 0.14 |
| Gyarmati | 2017 | Hungary | RCT | Mild to moderate OA | 23 | Hévíz mud applied directly to both hands, 3 weeks | EQ-5D | Patient | |  | 0.72 | 0.15 |
| Gyarmati | 2017 | Hungary | RCT | Mild to moderate OA | 23 | Hévíz mud applied directly to both hands, 4 months | EQ-5D | Patient | |  | 0.72 | 0.20 |
| Harijee | 2021 | India | Cross-sectional study | Bilateral hand amputation | 32 | Bilateral hand amputation- 6 months post-op | EQ‐5D‐5L | Patient | |  | 0.46 | 0.2 |
| Harijee | 2021 | India | Cross-sectional study | Bilateral hand amputation | 32 | Bilateral hand amputation- 6 months post-op | EQ-VAS | Patient | |  | 0.61 | 0.25 |
| Holmes | 2016 | UK | RCT | Scapholunate instability | 5 | Scapholunate instability, baseline | EQ-5D | Patient | |  | 0.71 | - |
| Holmes | 2016 | UK | RCT | Scapholunate instability | 5 | Scapholunate instability, 16 months post physiotherapy | EQ-5D | Patient | |  | 0.90 | - |
| Hustedt | 2023 | USA | Prospective cohort | Osteoarthritis | 34 | Base of thumb osteoarthritis without symptomatic improvement following non-operative management - baseline | EQ-5D | Patient | |  | 0.52 | 0.22 |
| Hustedt | 2023 | USA | Prospective cohort | Osteoarthritis | 34 | Base of thumb osteoarthritis without symptomatic improvement following non-operative management - 12 months following denervation | EQ-5D | Patient | |  | 0.82 | 0.24 |
| Hustedt | 2023 | USA | Prospective cohort | Osteoarthritis | 14 | Base of thumb osteoarthritis without symptomatic improvement following non-operative management - baseline | EQ-5D | Patient | |  | 0.44 | 0.15 |
| Hustedt | 2023 | USA | Prospective cohort | Osteoarthritis | 14 | Base of thumb osteoarthritis without symptomatic improvement following non-operative management - 12 months following suspension arthroplasty | EQ-5D | Patient | |  | 0.82 | 0.08 |
| Jerosch-Herold | 2017 | UK | Prospective cohort | CTS | 63 | Baseline, CTS-6 score 4.01-5.00 | EQ-5D |  | |  | 0.43 | 0.34 |
| Jerosch-Herold | 2017 | UK | Prospective cohort | CTS | 241 | Baseline, CTS-6 score 3.01-4.00 | EQ-5D |  | |  | 0.60 | 0.28 |
| Jerosch-Herold | 2017 | UK | Prospective cohort | CTS | 24 | Baseline, CTS NCS grade 6 | EQ-5D |  | |  | 0.61 | 0.26 |
| Jerosch-Herold | 2017 | UK | Prospective cohort | CTS | 150 | Baseline, CTS NCS grade 1 | EQ-5D |  | |  | 0.66 | 0.28 |
| Jerosch-Herold | 2017 | UK | Prospective cohort | CTS | 159 | Baseline, CTS NCS grade 5 | EQ-5D |  | |  | 0.66 | 0.24 |
| Jerosch-Herold | 2017 | UK | Prospective cohort | CTS | 79 | Baseline, CTS NCS grade 2 | EQ-5D |  | |  | 0.68 | 0.25 |
| Jerosch-Herold | 2017 | UK | Prospective cohort | CTS | 202 | Baseline, CTS NCS grade 3 | EQ-5D |  | |  | 0.68 | 0.27 |
| Jerosch-Herold | 2017 | UK | Prospective cohort | CTS | 137 | Baseline, CTS NCS grade 4 | EQ-5D |  | |  | 0.68 | 0.26 |
| Jerosch-Herold | 2017 | UK | Prospective cohort | CTS | 308 | Baseline, CTS-6 score 2.01-3.00 | EQ-5D |  | |  | 0.72 | 0.20 |
| Jerosch-Herold | 2017 | UK | Prospective cohort | CTS | 134 | Baseline, CTS-6 score 1.01-2.00 | EQ-5D |  | |  | 0.77 | 0.20 |
| Jerosch-Herold | 2017 | UK | Prospective cohort | CTS | 7 | Baseline, CTS-6 score 1.00 | EQ-5D |  | |  | 0.78 | 0.32 |
| Kazmers | 2016 | UK | Prospective cohort | Base of thumb OA | 14 | Arthrodesis, 6 weeks | EQ-5D |  | |  | 0.69 | - |
| Kazmers | 2016 | UK | Prospective cohort | Base of thumb OA | 14 | Arthrodesis, baseline | EQ-5D |  | |  | 0.73 | - |
| Kazmers | 2016 | UK | Prospective cohort | Base of thumb OA | 22 | LRTI, 6 weeks | EQ-5D |  | |  | 0.81 | - |
| Kazmers | 2016 | UK | Prospective cohort | Base of thumb OA | 22 | LRTI, baseline | EQ-5D |  | |  | 0.82 | - |
| Kerver | 2022 | Nederlands | Economic analysis | upper limb amputation | 28 | Amputee with multi-grip myoelectric prosthesis | EQ‐5D‐5L | Patient | |  | 0.80 | 0.21 |
| Kerver | 2022 | Nederlands | Economic analysis | upper limb amputation | 78 | Amputee with standard myoelectric prosthesis | EQ‐5D‐5L | Patient | |  | 0.84 | 0.17 |
| Kerver | 2022 | Nederlands | Economic analysis | upper limb amputation | 97 | Amputee with cosmetic prosthesis | EQ‐5D‐5L | Patient | |  | 0.86 | 0.15 |
| Kerver | 2022 | Nederlands | Economic analysis | upper limb amputation | 26 | Amputee with body-powered prosthesis | EQ‐5D‐5L | Patient | |  | 0.85 | 0.15 |
| Kerver | 2022 | Nederlands | Economic analysis | upper limb amputation | 13 | Amputee with prosthesis fitted with tools/accessories | EQ‐5D‐5L | Patient | |  | 0.72 | 0.22 |
| Kimpdf | 2021 | Korea | Prospective cohort | Osteoarthritis | 95 | Osteoarthritis of the hand | EQ-5D | Patient | | Korea | 0.82* |  |
| Konthal-de Bos | 2006 | Netherlands | RCT | CTS | 83 | Splint, 12 months | EQ-5D | Patient | |  | 0.81 | 0.16 |
| Konthal-de Bos | 2006 | Netherlands | RCT | CTS | 73 | Decompression, 12 months | EQ-5D | Patient | |  | 0.85 | 0.12 |
| Kosugi | 2021 | Japan | Retrospective cohort | Various musculoskeletal disorders of the hand | 1038 | Various musculoskeletal disorders of the hand | EQ-5D | Patient | |  | 0.66 |  |
| Kuboi | 2021 | Japan | Prospective cohort | Dupuytren's disease | 14 | Baseline | EQ-5D | Patient | | Japan | 0.81 | 0.21 |
| Kuboi | 2021 | Japan | Prospective cohort | Dupuytren's disease | 14 | 6 months post Clostridium histolyticum injection | EQ-5D | Patient | | Japan | 0.97 | 0.09 |
| Lane | 2020 | UK | cohort study | Base of thumb OA | 648 | Trapeziectomy with LRTI, 6 months | EQ-5D |  | |  | 0.66 | - |
| Lane | 2020 | UK | cohort study | Base of thumb OA | 749 | Trapeziectomy, 6 months | EQ-5D |  | |  | 0.69 | - |
| Marti | 2016 | switzerland | Prospective cohort | CTS | 60 | Baseline | EQ-5D |  | |  | 0.81 | - |
| Marti | 2016 | switzerland | Prospective cohort | CTS | 60 | Decompression, 6 months | EQ-5D |  | |  | 0.87 | - |
| MacEachen | 2022 | UK | Retrospective cohort | Hand amputation | 20 | Baseline | EQ-5D | Patient | | UK | 0.84* |  |
| MacEachen | 2022 | UK | Retrospective cohort | Hand amputation | 20 | 6 months post provision of multiarticulating hand prosthesis | EQ-5D | Patient | | UK | 0.84* |  |
| Millrose | 2020 | Germany | Retrospective cohort | CRPS | 21 | CRPS, physiotherapy 4 months | EQ-5D | Patient | |  | 0.66 | - |
| Millrose | 2020 | Germany | Retrospective cohort | CRPS | 21 | CRPS, baseline | EQ-5D | Patient | |  | 0.67 | - |
| Millrose | 2020 | Germany | Retrospective cohort | Wrist injury (various) | 24 | Wrist injury (various), baseline | EQ-5D | Patient | |  | 0.71 | - |
| Millrose | 2020 | Germany | Retrospective cohort | CRPS | 21 | CRPS, physiotherapy 1 week | EQ-5D | Patient | |  | 0.72 | - |
| Millrose | 2020 | Germany | Retrospective cohort | Wrist injury (various) | 24 | Wrist inury (various), physiotherapy 1 week | EQ-5D | Patient | |  | 0.76 | - |
| Millrose | 2020 | Germany | Retrospective cohort | Wrist injury (various) | 24 | Wrist injury (various), physiotherapy 4 months | EQ-5D | Patient | |  | 0.77 | - |
| Millrose | 2020 | Germany | Retrospective cohort | Finger injury (various) | 31 | Finger injury (various), baseline | EQ-5D | Patient | |  | 0.81 | - |
| Millrose | 2020 | Germany | Retrospective cohort | Finger injury (various) | 31 | Finger injury (various), 1 week | EQ-5D | Patient | |  | 0.86 | - |
| Millrose | 2020 | Germany | Retrospective cohort | Finger injury (various) | 31 | Finger injury (various), 4 months | EQ-5D | Patient | |  | 0.86 | - |
| Mosegaard | 2020 | Denmark | Retrospective cohort | CTS | 417 | CTS, baseline | EQ-5D | Patient | |  | 0.74 | - |
| Mosegaard | 2020 | Denmark | Retrospective cohort | CTS | 417 | CTS, 12 months post release (open or endoscopic) | EQ-5D | Patient | |  | 0.89 | - |
| Nazari | 2016 | Canada | Cross sectional | CTS | 22 | CTS (male), baseline | HUI-3 | Patient | |  | 0.58 | 0.24 |
| Nazari | 2016 | Canada | Cross sectional | CTS | 52 | CTS (female), baseline | HUI-3 | Patient | |  | 0.60 | 0.27 |
| Nazari | 2016 | Canada | Cross sectional | CTS | 52 | CTS (female), baseline | HUI-2 | Patient | |  | 0.71 | 0.20 |
| Nazari | 2016 | Canada | Cross sectional | CTS | 22 | CTS (male), baseline | HUI-2 | Patient | |  | 0.73 | 0.15 |
| Nazari | 2016 | Canada | Cross sectional | CTS | 52 | CTS (female), baseline | EQ-5D | Patient | |  | 0.74 | 0.18 |
| Nazari | 2016 | Canada | Cross sectional | CTS | 22 | CTS (male), baseline | EQ-5D | Patient | |  | 0.76 | 0.10 |
| Oppong | 2014 | UK | Economic evaluation | OA | 65 | Leaflet and advice, baseline | EQ-5D |  | |  | 0.62 | 0.26 |
| Oppong | 2014 | UK | Economic evaluation | OA | 65 | Leaflet and advice, 12 months | EQ-5D |  | |  | 0.63 | 0.22 |
| Oppong | 2014 | UK | Economic evaluation | OA | 62 | Joint protection only, 6 months | EQ-5D |  | |  | 0.64 | 0.25 |
| Oppong | 2014 | UK | Economic evaluation | OA | 65 | Hand exercises only, baseline | EQ-5D |  | |  | 0.65 | 0.21 |
| Oppong | 2014 | UK | Economic evaluation | OA | 62 | Joint protection only, baseline | EQ-5D |  | |  | 0.65 | 0.25 |
| Oppong | 2014 | UK | Economic evaluation | OA | 65 | Leaflet and advice, 6 months | EQ-5D |  | |  | 0.66 | 0.25 |
| Oppong | 2014 | UK | Economic evaluation | OA | 65 | Joint protection and hand exercise, baseline | EQ-5D |  | |  | 0.66 | 0.26 |
| Oppong | 2014 | UK | Economic evaluation | OA | 65 | joint protection and hand exercise, 12 months | EQ-5D |  | |  | 0.66 | 0.27 |
| Oppong | 2014 | UK | Economic evaluation | OA | 65 | Joint protection and hand exercise, 3 months | EQ-5D |  | |  | 0.66 | 0.22 |
| Oppong | 2014 | UK | Economic evaluation | OA | 65 | Leaflet and advice, 3 months | EQ-5D |  | |  | 0.67 | 0.24 |
| Oppong | 2014 | UK | Economic evaluation | OA | 65 | Joint protection and hand exercise, 6 months | EQ-5D |  | |  | 0.67 | 0.24 |
| Oppong | 2014 | UK | Economic evaluation | OA | 65 | Joint protection and hand exercise, 3 months | EQ-5D |  | |  | 0.68 | 0.24 |
| Oppong | 2014 | UK | Economic evaluation | OA | 62 | Joint protection only, 3 months | EQ-5D |  | |  | 0.68 | 0.17 |
| Oppong | 2014 | UK | Economic evaluation | OA | 62 | Joint protection only, 12 months | EQ-5D |  | |  | 0.68 | 0.19 |
| Oppong | 2014 | UK | Economic evaluation | OA | 65 | Hand exercises only, 6 months | EQ-5D |  | |  | 0.69 | 0.18 |
| Oppong | 2014 | UK | Economic evaluation | OA | 65 | Hand exercises only, 12 months | EQ-5D |  | |  | 0.71 | 0.18 |
| Ronaldson | 2021 | UK | Economic evaluation | OA | 123 | Baseline hydroxychloroquine | EQ-5D | Patient | | UK | 0.615 | 0.176 |
| Ronaldson | 2021 | UK | Economic evaluation | OA | 106 | 6 month post Hydroxychloroquine | EQ-5D | Patient | | UK | 0.635 | 0.198 |
| Ronaldson | 2021 | UK | Economic evaluation | OA | 90 | 12 month post Hydroxychloroquine | EQ-5D | Patient | | UK | 0.638 | 0.206 |
| Ronaldson | 2021 | UK | Economic evaluation | OA | 121 | Baseline placebo | EQ-5D | Patient | | UK | 0.612 | 0.18 |
| Ronaldson | 2021 | UK | Economic evaluation | OA | 103 | 6 month post placebo | EQ-5D | Patient | | UK | 0.641 | 0.171 |
| Ronaldson | 2021 | UK | Economic evaluation | OA | 97 | 12 month post placebo | EQ-5D | Patient | | UK | 0.642 | 0.204 |
| Rosberg | 2013 | Sweden | Prospective cohort | Composite serious hand and arm injuries | 45 | Composite major hand and arm injuries (amputation, spaghetti wrist, revascularisation, burn), 3 month | EQ-5D | Patient | |  | 0.70 | - |
| Rosberg | 2013 | Sweden | Prospective cohort | Composite serious hand and arm injuries | 45 | Composite major hand and arm injuries (amputation, spaghetti wrist, revascularisation, burn), 6 month | EQ-5D | Patient | |  | 0.75 | - |
| Rosberg | 2013 | Sweden | Prospective cohort | Composite serious hand and arm injuries | 45 | Composite major hand and arm injuries (amputation, spaghetti wrist, revascularisation, burn), 12 month | EQ-5D | Patient | |  | 0.80 | - |
| Rosberg | 2014 | Sweden | Retrospective cohort | Wrist replant/ revascularisation | 9 | Wrist replant/revascularisation | VAS | Patient | |  | 0.70 | - |
| Rosberg | 2014 | Sweden | Retrospective cohort | Wrist replant/ revascularisation | 9 | Wrist replant/revascularisation | EQ-5D | Patient | |  | 0.71 | - |
| Rosberg | 2014 | Sweden | Retrospective cohort | Total replant/ revascularisation | 326 | Replant/revascularisation at any level | VAS | Patient | |  | 0.80 | - |
| Rosberg | 2014 | Sweden | Retrospective cohort | Thumb replant/ revascularisation | 83 | Thumb replant | VAS | Patient | |  | 0.80 | - |
| Rosberg | 2014 | Sweden | Retrospective cohort | finger replant/ revascularisation | 205 | Finger replant | VAS | Patient | |  | 0.80 | - |
| Rosberg | 2014 | Sweden | Retrospective cohort | Carpus replant/ revascularisation | 14 | Replant at level of carpus | VAS | Patient | |  | 0.80 | - |
| Rosberg | 2014 | Sweden | Retrospective cohort | Wrist replant/ revascularisation | 13 | Replant proximal to wrist | VAS | Patient | |  | 0.80 | - |
| Rosberg | 2014 | Sweden | Retrospective cohort | Total replant/ revascularisation | 326 | Replant/revascularisation at any level | EQ-5D | Patient | |  | 0.82 | - |
| Rosberg | 2014 | Sweden | Retrospective cohort | Thumb replant/ revascularisation | 83 | Thumb replant | EQ-5D | Patient | |  | 0.82 | - |
| Rosberg | 2014 | Sweden | Retrospective cohort | Finger replant/ revascularisation | 205 | Finger replant | EQ-5D | Patient | |  | 0.82 | - |
| Rosberg | 2014 | Sweden | Retrospective cohort | Carpus replant/ revascularisation | 14 | Replant at level of carpus | EQ-5D | Patient | |  | 0.82 | - |
| Rosberg | 2014 | Sweden | Retrospective cohort | Wrist replant/ revascularisation | 13 | Replant proximal to wrist | EQ-5D | Patient | |  | 0.82 | - |
| Sauni | 2009 | Sweden | Retrospective cohort | Hand-arm vibration syndrome | 52 | Vibration induced white finger (symptoms improved) | EQ-5D | Patient | |  | 0.72 | - |
| Sauni | 2009 | Sweden | Retrospective cohort | Hand-arm vibration syndrome | 93 | Vibration induced white finger (symptoms stable or deteriorated) | EQ-5D | Patient | |  | 0.66 | - |
| Sauni | 2009 | Sweden | Retrospective cohort | Hand-arm vibration syndrome | 42 | Numbness or tingling of fingers (symptoms improved) | EQ-5D | Patient | |  | 0.76 | - |
| Sauni | 2009 | Sweden | Retrospective cohort | Hand-arm vibration syndrome | 102 | Numbness or tingling of fingers (symptoms stable or deteriorated) | EQ-5D | Patient | |  | 0.65 | - |
| Slatkowsky | 2007 | Norway | Prospective cohort | RA | 194 | Symptomatic rheumatoid arthritis of the hand | SF-6D | Patient | |  | 0.63 | 0.01 |
| Slatkowsky | 2007 | Norway | Prospective cohort | OA | 190 | Symptomatic osteoarthritis of the hand | SF-6D | Patient | |  | 0.64 | 0.01 |
| Stirling | 2020 | UK | Prospective cohort | Trigger finger | 192 | Baseline | EQ-5D | Patient | | UK | 0.77* | - |
| Stirling | 2020 | UK | Prospective cohort | Trigger finger | 192 | Steroid and pulley release, 13 months | EQ-5D | Patient | | UK | 0.80* | - |
| Tessler | 2017 | Canada | Retrospective cohort | Digital amputation | 15 | Digital amputation, more than 12 months | VAS | Patient | |  | 0.75 | - |
| Tessler | 2017 | Canada | Retrospective cohort | Digital replant | 36 | Digital replant, more than 12 months | VAS | Patient | |  | 0.84 | - |
| Thoma | 2004 | Canada | Prospective cohort | CTS | 6 | Open release, 6 weeks | HUI-3 | Patient | |  | 0.81 | 0.22 |
| Thoma | 2004 | Canada | Prospective cohort | CTS | 9 | Open release with novel ligament reconstruction, 6 weeks | HUI-3 | Patient | |  | 0.68 | 0.26 |
| Wang | 2020 | Canada | Economic evaluation | CTS | 11 | Severe CTS (BCTQ) | SF-6D | Patient | | Canada | 0.62 | 0.80 |
| Wang | 2020 | Canada | Economic evaluation | CTS | 11 | Severe CTS (BCTQ) | EQ-5D | Patient | | Canada | 0.63 | 0.29 |
| Wang | 2020 | Canada | Economic evaluation | CTS | 18 | Mild to moderate CTS (BCTQ) | SF-6D | Patient | | Canada | 0.72 | 0.19 |
| Wang | 2020 | Canada | Economic evaluation | CTS | 18 | Mild to moderate CTS (BCTQ) | VAS | Patient | | Canada | 0.75 | 0.25 |
| Wang | 2020 | Canada | Economic evaluation | CTS | 11 | Severe CTS (BCTQ) | VAS | Patient | | Canada | 0.80 | 0.19 |
| Wang | 2020 | Canada | Economic evaluation | CTS | 18 | Mild to moderate CTS (BCTQ) | EQ-5D | Patient | | Canada | 0.81 | 0.10 |
| Yeoman | 2018 | UK | Retrospective cohort | Base of thumb OA | 205 | Trapeziectomy, 8 years | EQ-5D | Patient | | UK | 0.56 | 0.31 |
| Yoon | 2019 | US/ Asia | Economic evaluation | Digital amputation | 47 | Single finger revision amputation (no thumb), 12 months | SF-6D | Patient | |  | 0.85 | - |
| Yoon | 2020 | US/ Asia | Economic evaluation | Digital amputation | 17 | Multi-finger revision amputation excluding thumb, 12 months | SF-6D | Patient | |  | 0.73 | - |
| Yoon | 2021 | US/ Asia | Economic evaluation | Digital amputation | 3 | Multi-finger including thumb revision amputation, 12 months | SF-6D | Patient | |  | 0.74 | - |
| Yoon | 2022 | US/ Asia | Economic evaluation | Digital amputation | 16 | Multi-finger replantation including thumb, 12 months | SF-6D | Patient | |  | 0.8 | - |
| Yoon | 2023 | US/ Asia | Economic evaluation | Digital amputation | 59 | Multi-finger replantation excluding thumb, 12 months | SF-6D | Patient | |  | 0.81 | - |
| Yoon | 2024 | US/ Asia | Economic evaluation | Digital amputation | 12 | Thumb only revision amputation, 12 months | SF-6D | Patient | |  | 0.82 | - |
| Yoon | 2025 | US/ Asia | Economic evaluation | Digital amputation | 36 | Thumb only replantation, 12 months | SF-6D | Patient | |  | 0.84 | - |
| Yoon | 2026 | US/ Asia | Economic evaluation | Digital amputation | 67 | Single finger replantation (no thumb), 12 months | SF-6D | Patient | |  | 0.86 | - |
| Zhang | 2021 | China | Retrospective cohort | Dupuytren's disease | 37 | Baseline | EQ-5D | Patient | |  | 0.72 | 0.28 |
| Zhang | 2021 | China | Retrospective cohort | Dupuytren's disease | 37 | Post ultrasound guided aponeurotomy | EQ-5D | Patient | |  | 0.88 | 0.72 |
| Zuang | 2020 | USA | Economic evaluation | Trigger finger | 5 | Baseline | VAS | Healthcare professionals | |  | 0.75 | 0.20 |
| BCTQ – Boston Carpal Tunnel Questionnaire; CTS – Carpal Tunnel Syndrome; CRPS – Complex Regional Pain Syndrome; DD – Dupuytren’s Disease; EQ-5D – EuroQol Five Dimensions; HUI-2 – Health Utility Index 2; HUI-3 Health Utility Index 3; LRTI – Ligament Reconstruction and Tendon Interposition; NCS – Nerve Conduction Study; OA – Osteoarthritis; ORIF – Open Reduction Internal Fixation; RA – Rheumatoid Arthritis; RCT – Randomised Control Trial; SF-6D – Short Form 6 Dimensions; VAS – Visual Analogue Scale  *Median utility value | | | | | | | | | | | | |

**Supplementary Table 2:** Catalogued average utility values derived using indirect valuation techniques with associated health states. Mean utility values (± standard deviation) are presented unless stated otherwise.


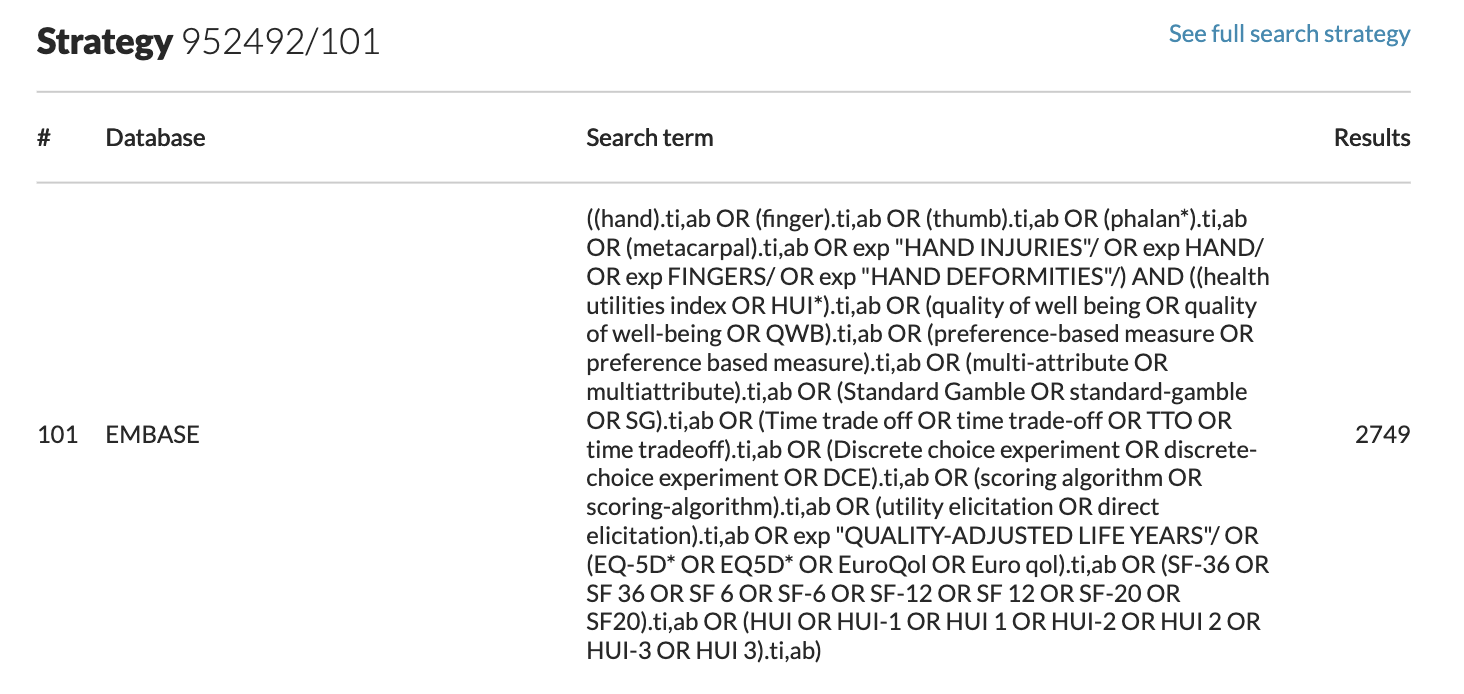


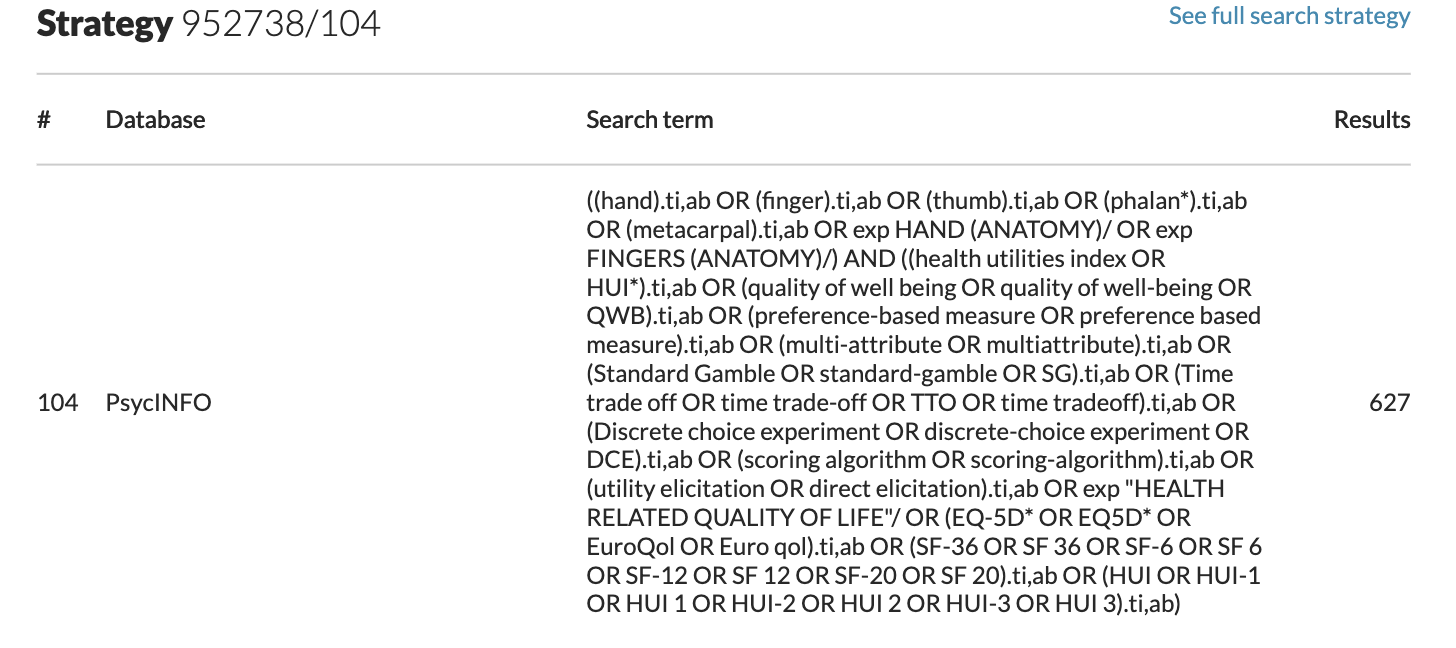


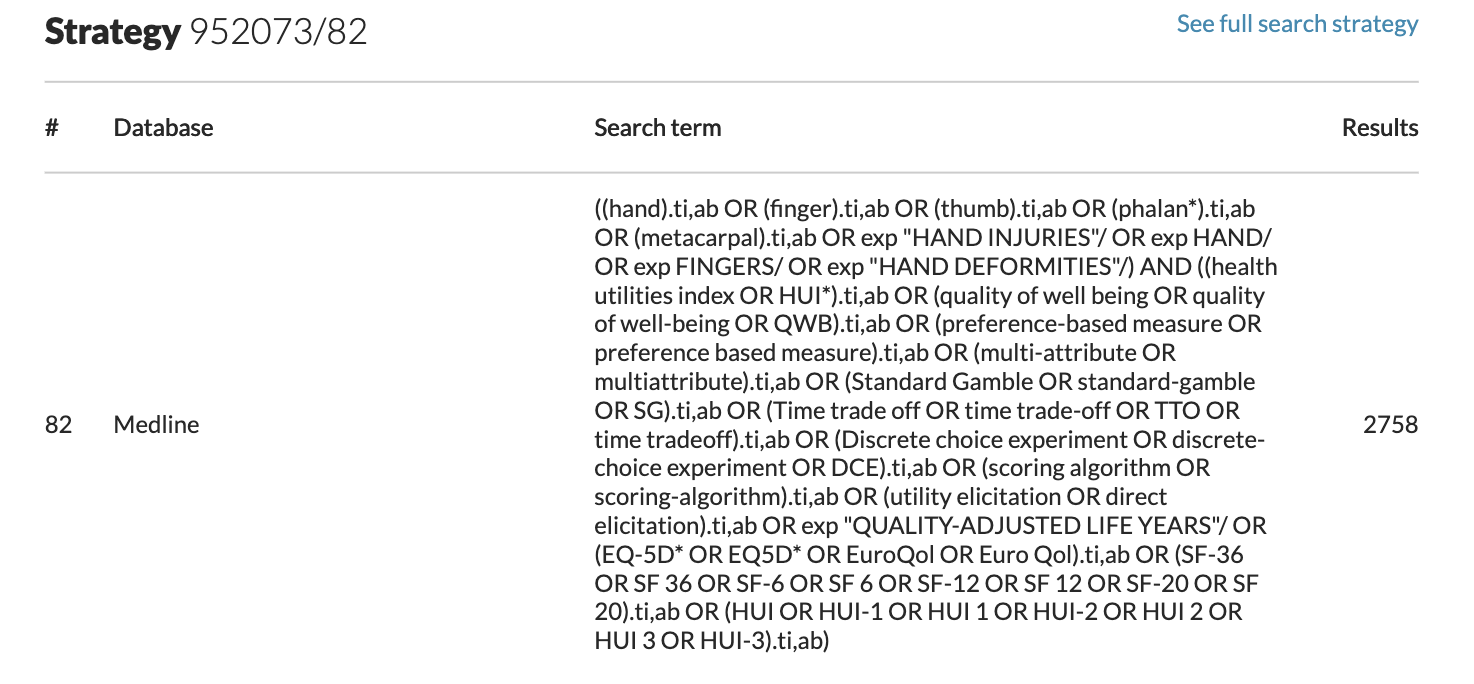


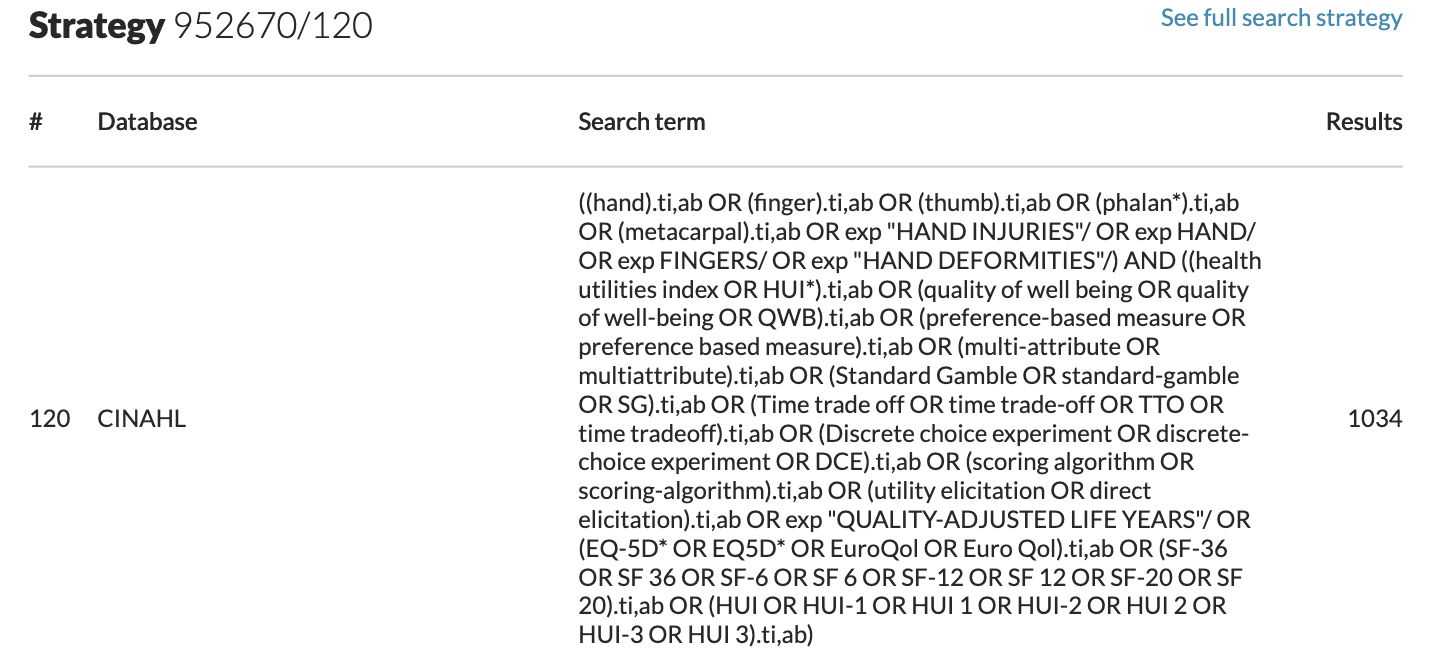


**CENTRAL**

ID Search Hits

#1 hand 34161

#2 finger 6496

#3 thumb 2093

#4 phalan* 356

#5 metacarpal 351

#6 MeSH descriptor: [Hand Injuries] explode all trees 275

#7 MeSH descriptor: [Hand] explode all trees 2307

#8 MeSH descriptor: [Fingers] explode all trees 836

#9 MeSH descriptor: [Hand Deformities] explode all trees 38

#10 health utilities index OR HUI* 5671

#11 quality of well being OR quality of well-being OR QWB 15596

#12 preference-based measure OR preference based measure 2439

#13 multi-attribute OR multiattribute 82

#14 Standard Gamble OR standard-gamble OR SG 9372

#15 Time trade off OR time trade-off OR TTO OR time tradeoff 1141

#16 Discrete choice experiment OR discrete-choice experiment OR DCE 539

#17 scoring algorithm OR scoring-algorithm 482

#18 utility elicitation OR direct elicitation 47

#19 MeSH descriptor: [Quality-Adjusted Life Years] explode all trees 1269

#20 EQ-5D* OR EQ5D* OR EuroQol OR Euro Qol 10119

#21 SF-36 OR SF 36 OR SF-6 OR SF 6 OR SF-12 OR SF 12 OR SF-20 OR SF 20 19975

#22 HUI OR HUI-1 OR HUI 1 OR HUI-2 OR HUI 2 OR HUI-3 OR HUI 3 1900

#23 #1 OR #2 OR #3 OR #4 OR #5 OR #6 OR #7 OR #8 OR #9 40120

#24 #10 OR #11 OR #12 OR #13 OR #14 OR #15 OR #16 OR #17 OR #18 OR #19 OR #20 OR #21 OR #22 56807

**Supplementary table 3:** Search strategies for specified databases
